# Supplementary material for: CDK1-Cyclin B1 Activates RNMT, Coordinating mRNA Cap Methylation with G1 Phase Transcription
Source: Mol Cell. 2016 Mar 3;61(5):734–46. doi: 10.1016/j.molcel.2016.02.008 (PMC4781437; doi:10.1016/j.molcel.2016.02.008)
Supplement: Document S1. Supplemental Experimental Procedures and Figures S1–S7 [file mmc1.pdf]

**Molecular Cell, Volume *61***

## Supplemental Information

# CDK1-Cyclin B1 Activates RNMT, Coordinating mRNA Cap Methylation with G1 Phase Transcription

**Michael Aregger, Aneesa Kaskar, Dhaval Varshney, Maria Elena Fernandez-Sanchez, Francisco A. Inesta-Vaquera, Simone Weidlich, and Victoria H. Cowling**

**Figure S1**

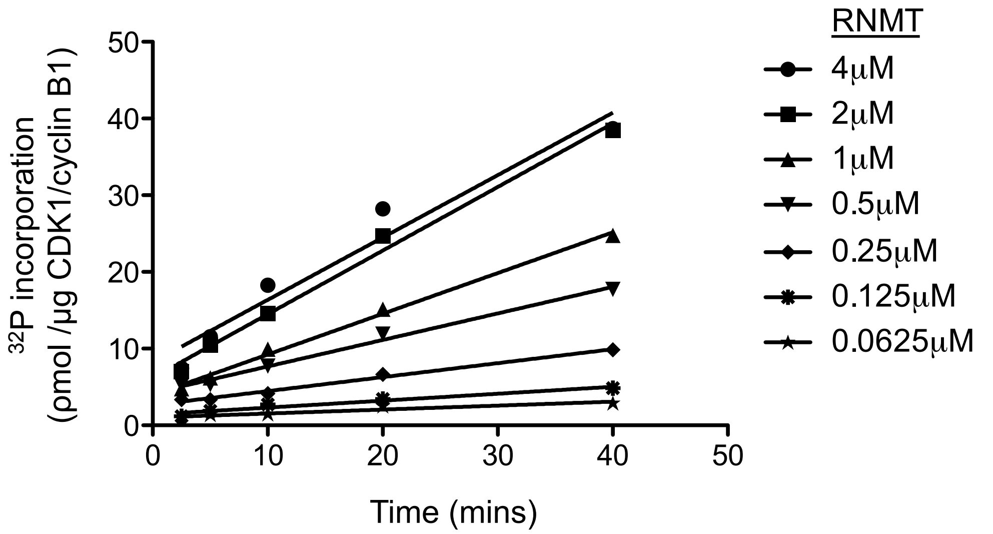


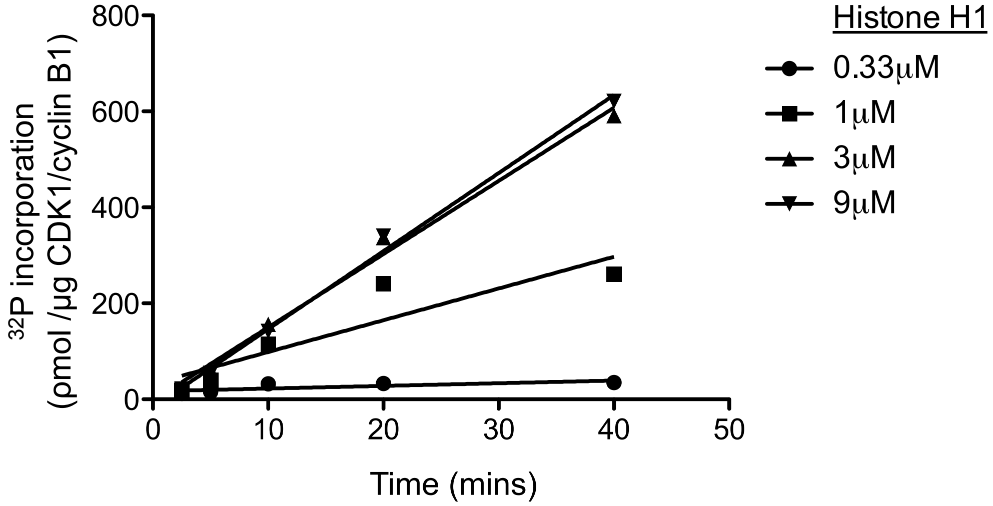


**Figure S1, related to Figure 3. CDK-cyclin B1 phosphorylation of RNMT and Histone H1**

0.01µM CDK1-cyclin B1 was incubated with a titration of RNMT or Histone H1 and $^{32}$P ATP for the time course indicated. Following the kinase reaction, proteins were resolved by SDS-PAGE and $^{32}$P-labelled protein was visualised by phosphoimaging and quantified by image densitometry.

**Figure S2**

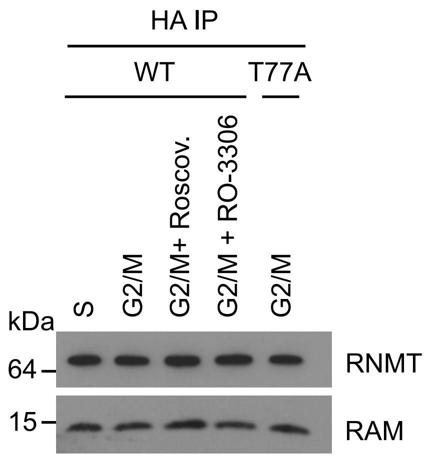


**Figure S2, related to Figure 4. RNMT phosphorylation does not impact on interaction with RAM**

HeLa cells stably expressing HA-RNMT WT or T77A were synchronised using a double thymidine block and released into normal growth media for 2 hours (S) or 7.5 hours (G2/M), or incubated for 7.5 hours and then for 15 minutes with 50 µM Roscovitine (G2/M + Roscov.) or 9 µM RO-3306 (G2/M + RO-3306). HA-RNMT was immunoprecipitated from 0.6 mg cell extracts using 7 µg anti-HA antibody. Immunoprecipitates were resolved by SDS-PAGE and WBs were performed to detect RNMT and RAM.

**Figure S3**

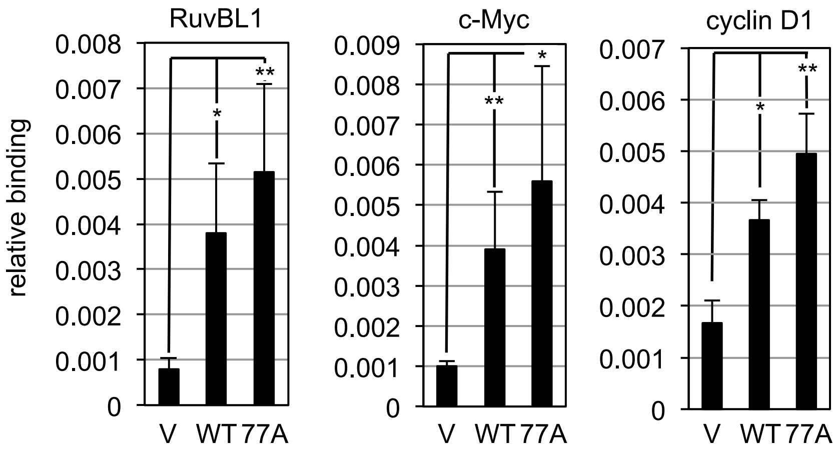


**Figure S3, related to Figure 4. Prevention of RNMT T77 phosphorylation does not alter RNMT recruitment to transcription initiation sites**

Chromatin immunoprecipitation was performed on HeLa cells transiently transfected with pcDNA4 HA-RNMT WT or T77A or with pcDNA4 (V). Anti-HA antibodies were used to immunoprecipitate RNMT. PCR analysis was performed against promoter proximal regions of the genes indicated. The PCR signal relative to input is depicted for the average of at least three independent experiments and the error bars represent the standard deviation (T-test P-value “*”>0.05, “**”>0.01).

**Figure S4**

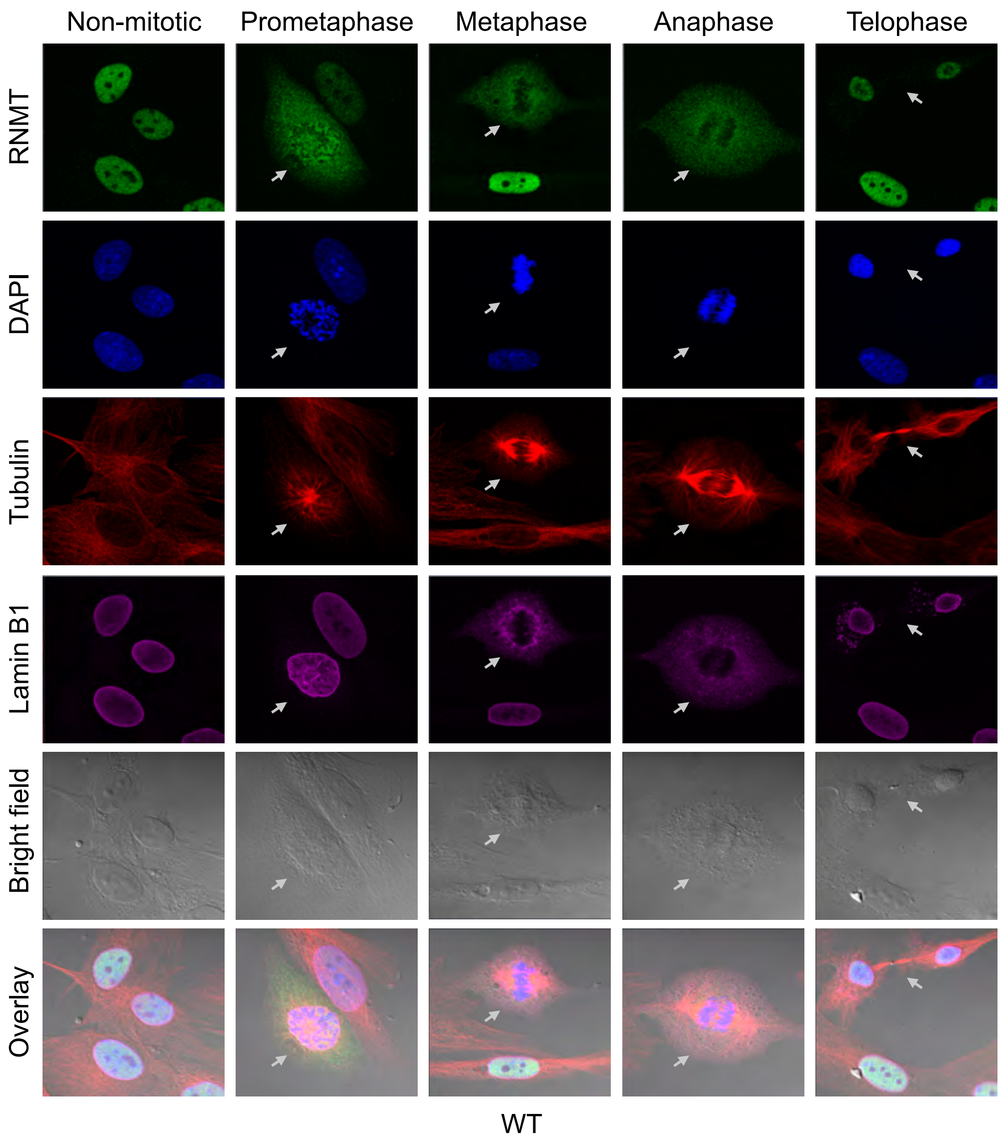


**Figure S4, related to Figure 5. RNMT Threonine 77 phosphorylation does not regulate RNMT localisation during mitosis**

IMEC cells expressing siRNA-resistant HA-RNMT WT were transfected with siRNMT for 72 hours and immunofluorescence analysis was performed.

**Figure S5**

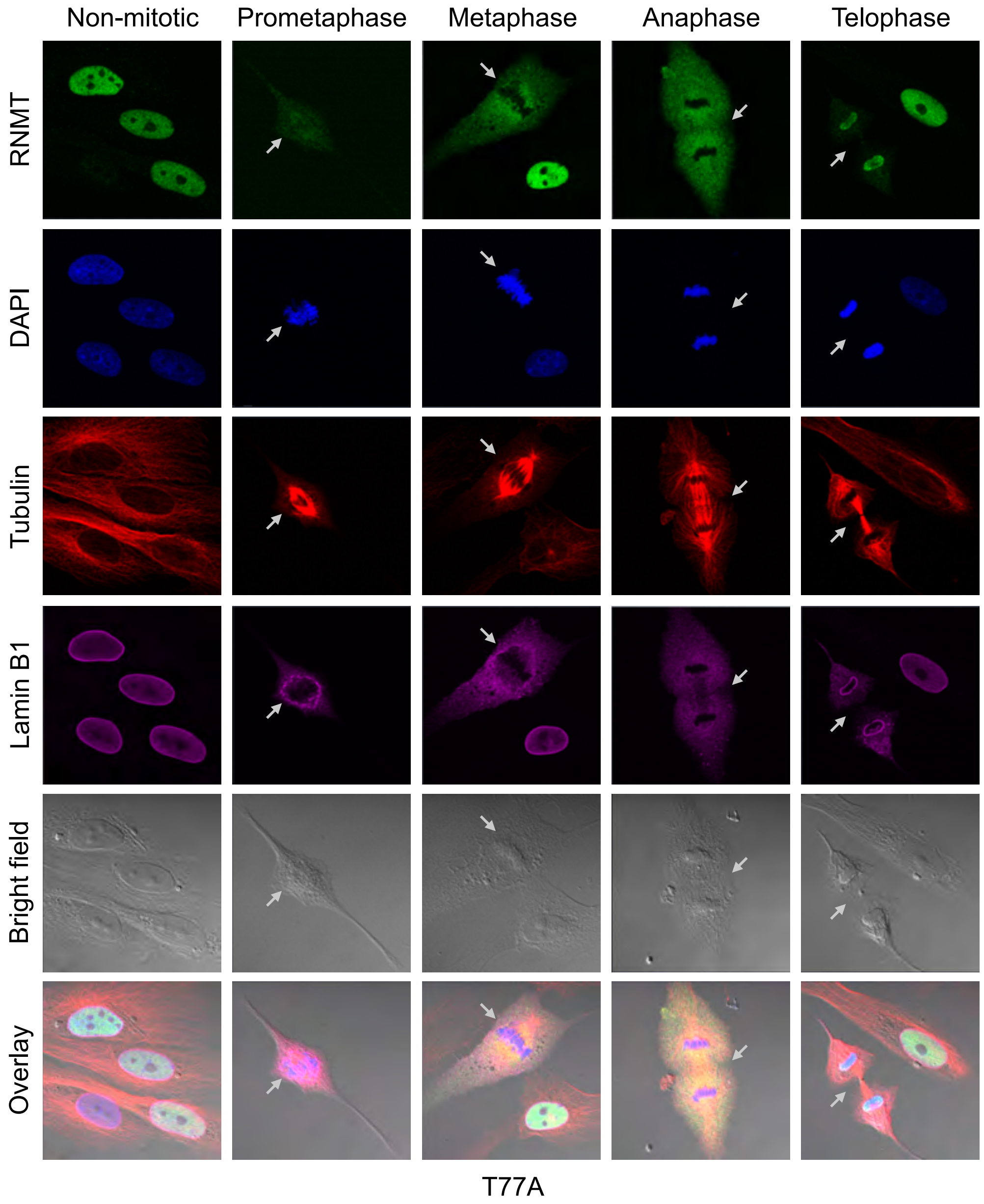


**Figure S5, related to Figure 5. RNMT T77 phosphorylation does not regulate RNMT localisation during mitosis**

IMEC cells expressing siRNA-resistant HA-RNMT T77A were transfected with siRNMT for 72 hours and immunofluorescence analysis was performed.

**Figure S6**

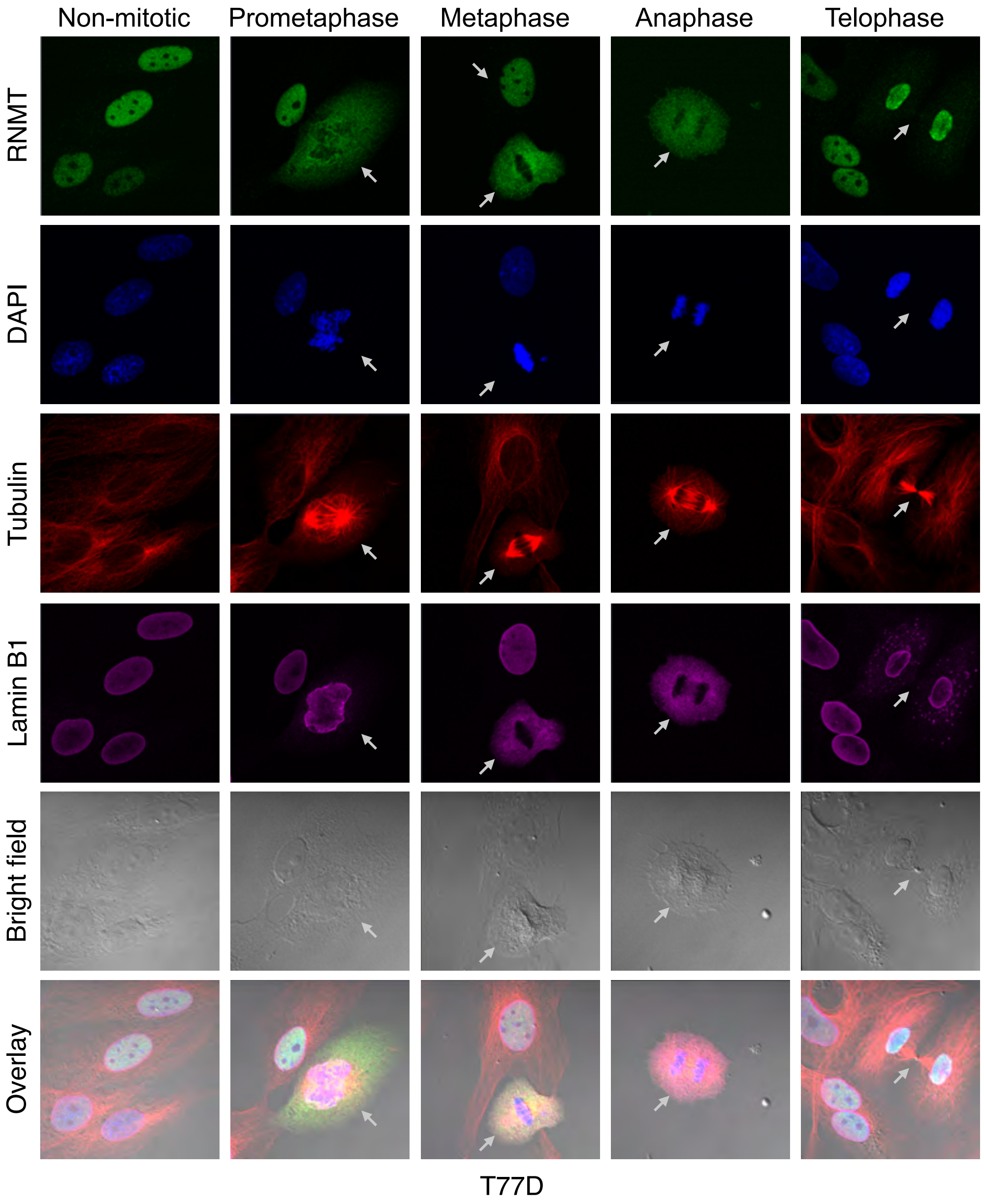


**Figure S6, related to Figure 5. RNMT T77 phosphorylation does not regulate RNMT localisation during mitosis**

IMEC cells expressing siRNA-resistant HA-RNMT T77D were transfected with siRNMT for 72 hours and immunofluorescence analysis was performed.

## Figure S7

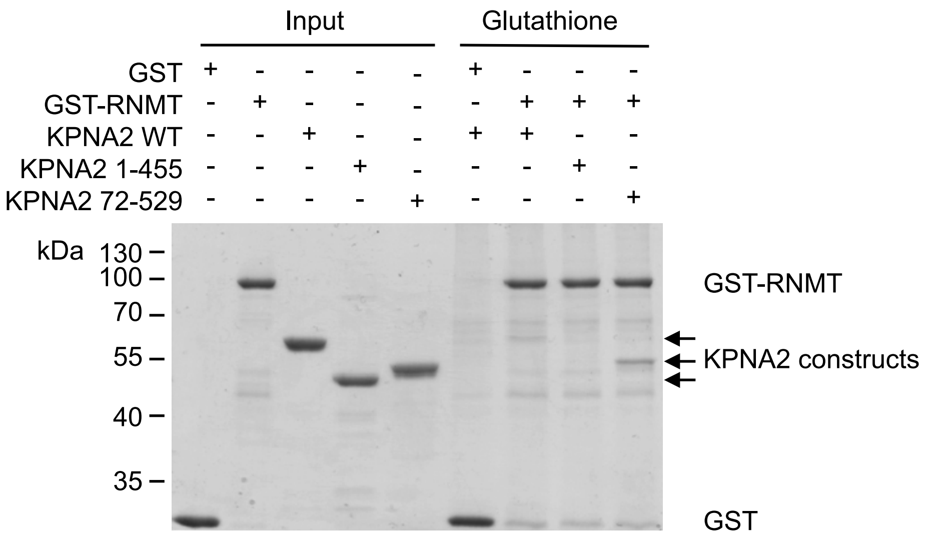


**Figure S7, related to Figure 5. RNMT directly interacts with KPNA2 WT and 72-529**

Recombinant GST-RNMT and GST were incubated with recombinant KPNA2 WT, 1-455, 72-529 and complexes affinity purified with glutathione-sepharose. Inputs and proteins eluted were resolved by SDS-PAGE and stained by Coomassie blue.

**Table S1, related to Figure 6. Summary of proteins identified by Mass spectrometry.**
Sequence coverage for mass spectrometry analysis of HeLa cells expressing HA-RNMT WT and HA-RNMT T77A.

**Table S2, related to Figure 6. Differential expression analysis of HeLa cells expressing HA-RNMT WT and 77A.**
Label Free Quantitation (LFQ) of proteins identified in 3 preparations of HeLa cells expressing HA-RNMT WT and T77A were compared. Proteins that were expressed with a significant difference in the two sample sets are indicated. Ttest P values reported.

**Table S3, related to Figure 6. GO Term analysis of proteins expressed significantly differently in response to HA-RNMT WT and 77A.**

**Supplemental Experimental Procedures**

**Cell Culture**
HeLa and HEK 293 cells were cultured in DMEM/10% Foetal Bovine Serum (GIBCO), in 5% $CO_2$ at 37ºC. IMEC (Human immortalised mammary epithelial cells) were cultured in DMEM/F-12 HAM supplemented with L-glutamine, 5 µg/ml insulin, 10 ng/ml EGF and 0.5 µg/ml hydrocortisone. Stable cell lines expressing INI (vector control) and INI-HA-RNMT constructs were generated by retroviral infection using standard protocols and selected with 0.5 mg/ml G418. siRNAs and controls were purchased from the siGenome range (Dharmacon). 3 µl Gene Juice (Novagen) was used to transfect 1.2 $x10^5$ cells with 1 µg DNA, 4 µl Lipofectamine RNAiMax (Invitrogen) was used to transfect 1.2 $x10^5$ cells with 4 µl 50µM siRNA, and Lipofectamine 2000 (Invitrogen) was used to simultaneously transfect 2.5 $x10^5$ cells with 4 µl siRNA and 1 µg DNA. Cells were lysed in Triton lysis buffer (10 mM Tris [pH 7.05], 50 mM NaCl, 50 mM NaF, 10% glycerol, 0.5% Triton X-100, protease and phosphatase inhibitors (Sigma)). Kinase inhibitors used were Roscovitine (Sigma), RO-3306 (Merck), DRB (Sigma) and Flavopiridol (Cayman). Soft agar assay: 5 x $10^5$ IMEC cells were plated in 0.3% noble agar/5% serum/growth medium and fed every 2 days.

**Orthophosphate Labelling**
HEK 293 cells were washed with PBS, incubated at 37°C in phosphate-deficient DMEM/10% dialysed FBS for 4 h,then labelled with 0.03 mCi/ml $^{32}$P orthophosphate for 2 h. 2.5 mg cell extract was precleared for 30 min with 30 µl protein A/G PLUS agarose and HA-RNMT immunoprecipitated for 4 h using 10 µg monoclonal anti-HA antibody-conjugated agarose (Sigma). Immunoprecipitates were washed with Triton lysis buffer and resuspended in 50 µl Lamelli buffer. 24 µl was resolved by SDS-PAGE and analysed by autoradiography and 5 µl was used for western blot analysis.

**Immunoprecipitation**
Performed at 4ºC. RNMT phosphorylation analysis: RNMT was immunoprecipitated from 600 µg HeLa cell extracts for 2.5 hours, using 6 µg mouse monoclonal anti-HA antibody-conjugated agarose or 1.5 µg sheep polyclonal anti-RNMT plus 20 µl Protein A/G agarose. Immunoprecipitates were washed and resolved by 8% SDS-PAGE. 50% of immunoprecipitates were used to detect pT77 RNMT and 10% used to detect RNMT. Other immunoprecipitations were performed with 1.5µg antibody and 1-5mg cell extract.

**m7G immunoprecipitation**
Experiments performed essentially as in (Cole and Cowling, 2009). HeLa cells were transfected with pcDNA5 HA-RNMT WT, 77A or vector control for 24 hrs. RNA was extracted and repurified by phenol:chloroform extraction. 5 µg was subject to immunoprecipitation with 10 µl anti-m7G antibody or polyclonal control rabbit antibody for 1 hr at room temperature. Immunoprecipitates were washed three times and resultant RNA used as a template for RTPCR.

**Peptide pull down**
Performed at 4 °C. 10 mg cell extracts were precleared five times for 10 minutes with 20 µl 50 % streptavidin-sepharose, incubated with 1-3 µg biotinylated peptides for 10 minutes and 20 µl 50 % streptavidine-sepharose for 5 minutes. Sepharose was washed three times with Triton Lysis buffer, proteins resolved by SDS-PAGE and identified by western blot or peptide mass fingerprinting. Protein was trypsinised and peptides resolved by nanoLC system coupled to LTQ-Orbitrap mass spectrometer. Results were searched against the SwissProt or IPI human databases using the Mascot Daemon. Mass Spec performed by MRC PPU mass spectrometry team.

**GST pull down**
Recombinant proteins including GST fusions were incubated for 3 hours at 4 °C, affinity purified with 25 µl 50% slurry glutathione-sepharose, washed five times with Triton Lysis Buffer and resolved by SDS-PAGE.

**Flow cytometry analysis**
HeLa cells were fixed by dropwise addition of cold Ethanol to 70% concentration. Cells were washed twice in PBS/1 % FBS and stained in PI solution (PBS, 1 % FBS, 50 µg/ml RNase A, 50 µg/ml propidium iodide) for 30 minutes at room temperature in the dark. DNA content was assessed using a FACS Calibur flow cytometry (Becton Dickinson). 10,000 events were counted per sample and DNA content was plotted on a histogram from channel number 0 to 1023 using the FlowJo software. The G1 peak was set to 200 to allow resolution of the G1, S and G2/M phases. Cell cycle distribution was estimated with FlowJo using the Watson distribution model

**Immunofluorescence analysis**
IMEC were seeded on cover slips and fixed with 4% paraformaldehyde in PHEM for 7 minutes at 37°C. Cells were permeabilised with 0.02 % Triton X-100 in TBST for 2.5 minutes and blocked with 10 % Donkey serum in antibody dilution solution (PBS, 0.2% BSA, 0.02% sodium azide) for 30 minutes. Cells were incubated with anti-HA (in house, 1:2,000), anti-Tubulin (Abcam,1:500) and anti-Lamin (Abcam, 1:1,000) antibodies for 1 hour at room temperature. Subsequently, cells were incubated with Alexa Fluor 488 Donkey anti-Sheep, Alexa Fluor 594 Donkey anti-Rat and Alexa 642 Donkey anti-Rabbit secondary antibodies (all Invitrogen, 1:500) for 50 minutes in the dark and stained with DAPI (Invitrogen, 1:50,000). Fluorescence microscopy was performed on a Zeiss LSM 700.

**Western blotting**
Western blots were performed according to standard protocols. Antibodies were raised against recombinant human RNMT, human RAM, and GST in sheep and affinity purified. Antibodies also used: monoclonal anti-HA (Sigma), and anti-FLAG (Sigma) antibodies, and polyclonal anti-Tubulin (Santa Cruz), Cyclin B1 (CST), Histone 3 pS10 (Abcam), Cyclin E1/2 (SCBT), Cyclin A2 (SCBT), CDK1 (CST), CDK3 (BIOSS), β-Tubulin (Santa Cruz), Tomm70A (Insight), Bop1 (Insight), DDX18 (Insight), Skp1 (SCBT) and KPNA2 (Thermo) antibodies. RNMT Phospho-T77 antibody production: antibodies were raised in sheep against the phosphopeptide RNMT 73CGKDT*PSKKR82 (T* indicates phospho-threonine) coupled separately to keyhole limpet hemocyanin and bovine serum albumin. Sera were purified by affinity chromatography on immobilized peptide antigen.

**Chromatin immunoprecipitations (ChIP)**
Chromatin immunoprecipitations were performed using the Millipore ChIP kit according to manufacturer's instructions. 15 μg anti-HA-agarose (Sigma) was used to immunoprecipitate HA-RNMT. The DNA was purified with a QIAquick PCR purification kit (Qiagen) and eluted in 50 μl distilled water. 2 μl of input and immunoprecipitated DNA were subjected to RT-PCR analysis. ChIP signal was determined relative to input.

**RTPCR analysis**
RTPCR performed with a BioRad iQ5 RT-PCR detection system using Quanta Biosciences SYBR Green FastMix for iQ and the primers listed below. Primers used were:

| | | |
|---|---|---|
| BOP1 | GTGGGCTTCAACCCCTATGAG | CCATGCGAGAGACCTTCTCC |
| Tomm70A | CCATATACCTGTGGAGTCGGC | GAGAGTTCATGTCCAAGTGAGCA |
| DDX18 | GGAAGGCAGGGATCTTCTAGC | TCCATTCCTGGGCATGAACC |
| Skp2 | ATGCCCCAATCTTGTCCATCT | CACCGACTGAGTGATAGGTGT |
| RuvBL1-chIP | TGTGGCCAGTGGACC | ACTTCCCTGAGGAAATAATGG |
| c-Myc-chIP | GCACTGGAACTTACAACACC | ATCCAGCGTCTAAGCAGC |
| cyclin D1-chIP | AGCTGCCCAGGAAGAGC | CCGCCTTCAGCATGG |

**Proteomic analysis**
HeLa cells were lysed in 8M urea/100 mM Tris-HCl (pH 8.0)/protease and phosphatase inhibitors (Roche), 10mM DTT added for 30 mins at 30°C, then 50 mM carboxyamidomethylated iodoacetamide for 45 mins at room temperature in dark. Urea concentration was adjusted to 0.8 M in Tris/$CaCl_2$ buffer before adding Trypsin Gold (Wako) to a substrate: trypsin ratio of 100:1 (w/w), and incubated at 30°C overnight. Peptides were separated into 10 fractions on hydrophilic strong anion exchange column and analysed on Ultimate 3000 RSLCnano system coupled to a LTQ OrbiTrap Velos Pro (both Thermo Scientific). MaxQuant was used to analyse RAW data using *H. sapiens* Uniprot database using a false discovery rate of 1%. Contaminant, reverse and only identified by site entries were removed. For the three replicates, average LFQ intensity and Student T-test P-value was calculated (unpaired, two-tailed, unequal variance). Gene ontology analysis was carried out using REVIGO (Supek et al., 2011).
