# Supplementary material for: CDK1-Cyclin B1 Activates RNMT, Coordinating mRNA Cap Methylation with G1 Phase Transcription
Source: Mol Cell. 2016 Mar 3;61(5):734–46. doi: 10.1016/j.molcel.2016.02.008 (PMC4781437; doi:10.1016/j.molcel.2016.02.008)
Supplement: Document S2. Article plus Supplemental Information [file mmc5.pdf]

Article

Molecular Cell

CDK1-Cyclin B1 Activates RNMT, Coordinating mRNA Cap Methylation with G1 Phase Transcription

Graphical Abstract

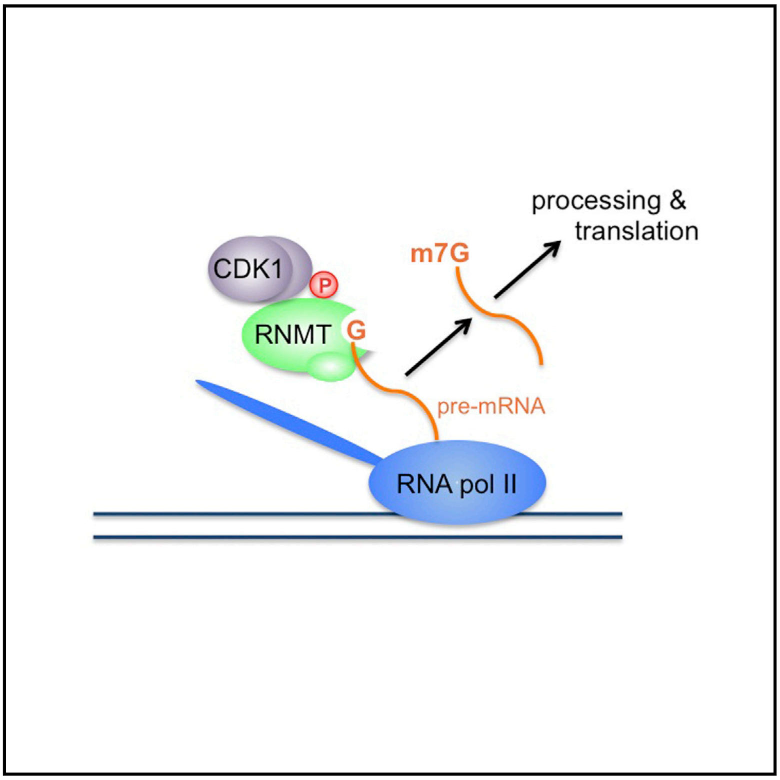

Highlights

- CDK1-cyclin B1 phosphorylates the mRNA cap methyltransferase RNMT on T77
- RNMT T77 phosphorylation increases mRNA cap methyltransferase activity
- CDK1-cyclin B1 activation of RNMT coordinates post-mitotic transcription and capping
- RNMT T77 phosphorylation is rate limiting for gene expression and cell proliferation

Authors

Michael Aregger, Aneesa Kaskar, Dhaval Varshney, Maria Elena Fernandez-Sanchez, Francisco A. Inesta-Vaquera, Simone Weidlich, Victoria H. Cowling

Correspondence

v.h.cowling@dundee.ac.uk

In Brief

mRNA processing and translation are dependent on the mRNA cap structure. Aregger et al. report that the cell cycle regulator CDK1-cyclin B1 phosphorylates and activates the mRNA cap methyltransferase RNMT, resulting in increased capping activity following mitosis. RNMT phosphorylation coordinates G1 phase transcription and the cap formation required for translation initiation.

Aregger et al., 2016, Molecular Cell *61*, 734–746
March 3, 2016 ©2016 The Authors
http://dx.doi.org/10.1016/j.molcel.2016.02.008

CDK1-Cyclin B1 Activates RNMT, Coordinating mRNA Cap Methylation with G1 Phase Transcription

Michael Aregger,[1,3] Aneesa Kaskar,[1] Dhaval Varshney,[1] Maria Elena Fernandez-Sanchez,[1,4] Francisco A. Inesta-Vaquera,[1] Simone Weidlich,[2] and Victoria H. Cowling[1,*]
[1]Centre for Gene Regulation and Expression/MRC Phosphorylation and Ubiquitylation, School of Life Sciences, University of Dundee, Dundee DD1 5EH, UK
[2]Division of Signal Transduction Therapy, University of Dundee, Dundee DD1 5EH, UK
[3]Present address: Donnelly Centre for Cellular and Biomolecular Research, University of Toronto, Toronto, ON M5S 3E1, Canada
[4]Present address: Institut Curie, Centre de Recherche, Unité Physicochimie Curie, Unités Mixtes des Recherches 168, 75005 Paris, France
*Correspondence: v.h.cowling@dundee.ac.uk
http://dx.doi.org/10.1016/j.molcel.2016.02.008
This is an open access article under the CC BY license (http://creativecommons.org/licenses/by/4.0/).

SUMMARY

The creation of translation-competent mRNA is dependent on RNA polymerase II transcripts being modified by addition of the 7-methylguanosine (m7G) cap. The factors that mediate splicing, nuclear export, and translation initiation are recruited to the transcript via the cap. The cap structure is formed by several activities and completed by RNMT (RNA guanine-7 methyltransferase), which catalyzes N7 methylation of the cap guanosine. We report that CDK1-cyclin B1 phosphorylates the RNMT regulatory domain on T77 during G2/M phase of the cell cycle. RNMT T77 phosphorylation activates the enzyme both directly and indirectly by inhibiting interaction with KPNA2, an RNMT inhibitor. RNMT T77 phosphorylation results in elevated m7G cap methyltransferase activity at the beginning of G1 phase, coordinating mRNA capping with the burst of transcription that occurs following nuclear envelope reformation. RNMT T77 phosphorylation is required for the production of cohort of proteins, and inhibiting T77 phosphorylation reduces the cell proliferation rate.

INTRODUCTION

Maturation of RNA polymerase (pol) II transcripts into translation-competent mRNA is dependent on addition of the 7-methylguanosine (m7G) cap to the first transcribed nucleotide (Shuman, 2015; Topisirovic et al., 2011). The resultant m7G cap protects transcripts from exonucleases and is a docking site for the cap binding complex (CBC), eukaryotic initiation factor 4F (eIF4F), and other complexes that mediate transcript splicing, polyadenylation, nuclear export, and translation initiation (Gonatopoulos-Pournatzis and Cowling, 2014; Topisirovic et al., 2011). The enzymes that catalyze m7G synthesis can also promote transcription by direct interactions with RNA pol II-associated factors (Buratowski, 2009; Perales and Bentley, 2009).

mRNA capping occurs during the early stages of transcription, allowing the cap structure to coordinate almost all mRNA processing events. RNA is synthesized with a 5′ triphosphate group on the first transcribed nucleotide on which the cap is formed by the sequential action of a triphosphatase, guanylyltransferase, and methyltransferase (Shuman, 2015). In mammals, RNA guanylyltransferase and 5′ triphosphatase (RNGTT) contains the triphosphatase and guanylyltransferase activities. The methyltransferase activity resides in a separate complex, RNMT-RAM, which consists of a catalytic subunit, RNA guanine-7 methyltransferase (RNMT), and an activating subunit, RNMT-activating mini-protein (RAM) (Gonatopoulos-Pournatzis et al., 2011; Pillutla et al., 1998; Tsukamoto et al., 1998). RNMT contains a regulatory domain that affects catalytic activity and recruits RNMT-RAM to transcription initiation sites (Aregger and Cowling, 2013).

Co-transcriptional capping is facilitated by the recruitment of the capping enzymes to the phosphorylated RNA pol II C-terminal domain (CTD) during the early stages of transcription (Buratowski, 2009; Nilson et al., 2015; Perales and Bentley, 2009). RNGTT is activated by direct interaction with phosphorylated RNA pol II (Ghosh et al., 2011) and elongation factor Spt5 (Wen and Shatkin, 1999). RNMT-RAM is also recruited to RNA pol II in a phosphorylation-dependent manner, although the interaction is unlikely to be direct because it cannot be observed with purified proteins in vitro (Aregger and Cowling, 2012, 2013). RNMT has been observed to be activated by interaction with Importin α-1 (KPNA2) (Wen and Shatkin, 2000).

The mRNA cap is a potent modification, and its regulation has significant effects on gene expression and cell physiology. In mammals, formation of the mRNA cap is regulated by c-Myc, a transcription factor oncogene that promotes deregulated cell proliferation in many human tumor types. Regulated capping is critical for c-Myc-dependent protein synthesis, cell proliferation, and transformation. c-Myc increases promoter recruitment of the kinase transcription factor II H (TFIIH), resulting in increased RNA pol II phosphorylation, which increases recruitment of the capping enzymes and elevated mRNA cap methylation (Aregger and Cowling, 2012; Cole and Cowling, 2009; Cowling and Cole, 2007). c-Myc also upregulates S-adenosyl homocysteine hydrolase (SAAH), which hydrolyses the repressive methylation by-product S-adenosyl homocysteine (SAH) (Fernandez-Sanchez

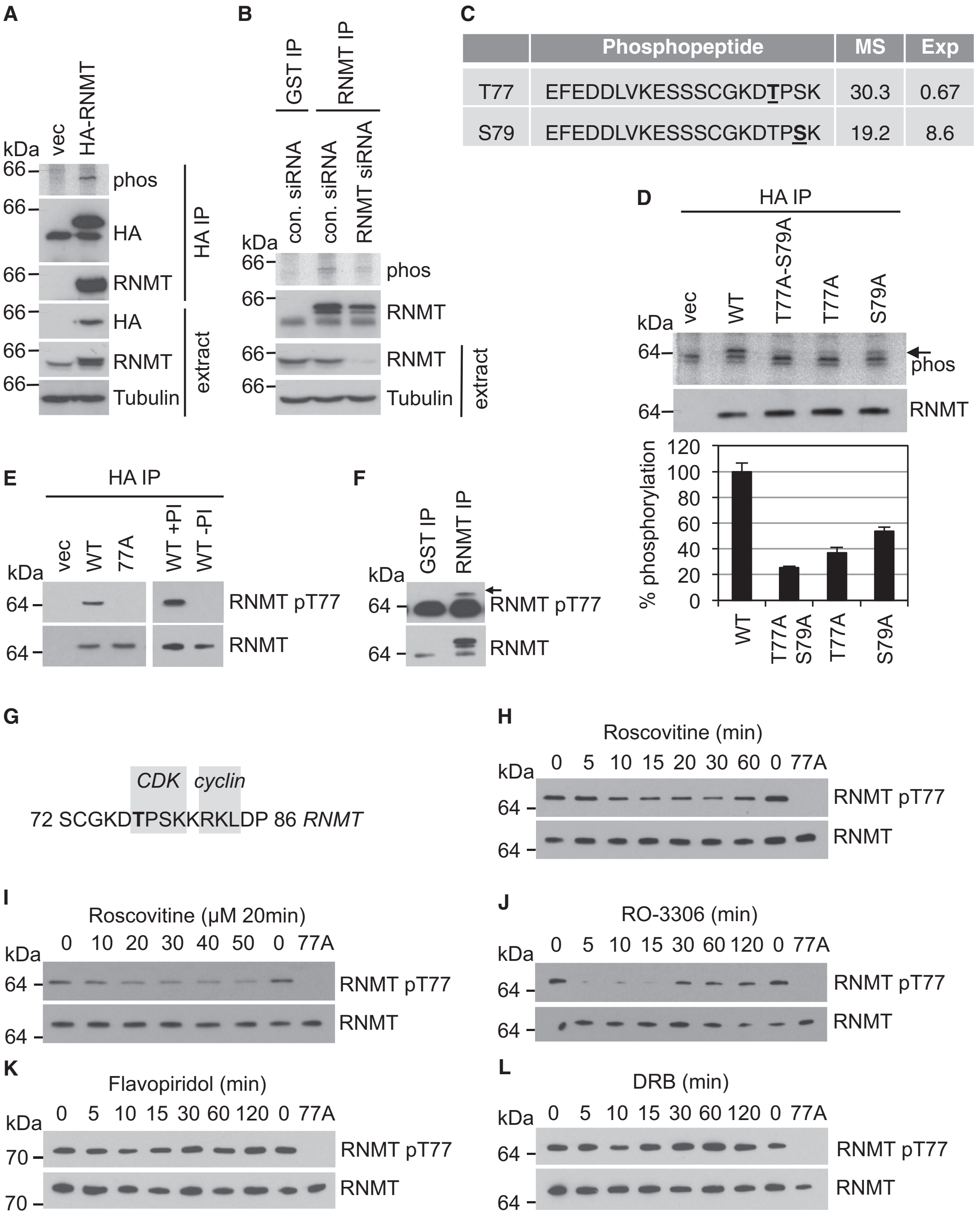

Figure 1. RNMT Is Phosphorylated on T77

(A) HA-RNMT-expressing 293 cells were cultured with ^{32}P orthophosphate, HA-RNMT-immunoprecipitated via an HA tag, and resolved by SDS-PAGE, and proteins were detected by phosphoimaging (phos) and western blot (WB). vec, vector.

(B) Cells were transfected with RNMT or control (con) siRNA for 48 hr and then cultured with ^{32}P orthophosphate. Endogenous RNMT IP was performed using anti-RNMT antibodies, and protein was detected. Anti-GST antibodies were used as a control.

(C) A phosphorylated RNMT peptide was identified in HA-RNMT by LC-MS/MS precursor ion scan. Potential phosphorylated amino acids are underlined. Mascot score (MS) and expected value (Exp) are indicated.

(D) HeLa cells expressing HA-RNMT WT, T77A-S79A, T77A, and S79A were cultured with ^{32}P orthophosphate and analyzed as in (A). Phospho-RNMT in two independent experiments was quantified relative to RNMT WT. Average and SD are depicted.

(E) WBs were performed to detect pT77 and total RNMT in HA-RNMT WT and T77A immunoprecipitates with (+PI) and without (−PI) phosphatase inhibitors.

(legend continued on next page)

et al., 2009). Another transcription factor, E2F1, has been found to promote RNA pol II phosphorylation and cap formation (Aregger and Cowling, 2012). Regulation of mRNA capping is also observed in yeast. Amino acid or glucose starvation reduces mRNA cap methylation (Jiao et al., 2010).

In this study we investigated whether signaling pathways that result in post-translational modification of the capping enzymes can directly regulate their activity. We report that RNMT is phosphorylated and activated by CDK1-cyclin B1, resulting in elevated cap methyltransferase activity when transcription is reinitiating after mitosis. RNMT phosphorylation is rate-limiting for gene expression and cell proliferation.

RESULTS

RNMT Is Phosphorylated on T77

To investigate RNMT phosphorylation, HEK293 cells expressing hemagglutinin (HA)-RNMT or vector control were cultured in ^{32}P orthophosphate, which is incorporated into cellular proteins. HA-RNMT was immunoprecipitated from cell extracts and resolved by gel electrophoresis, and a 65-kDa band consistent with the size of RNMT was detected by phosphoimaging, confirming phosphorylation of RNMT (Figure 1A). Endogenous RNMT phosphorylation was observed by the same methodology, the signal for which was reduced following transfection with RNMT small interfering RNA (siRNA) (Figure 1B). Mass spectrometric analysis of HA-RNMT purified from HeLa cells identified a peptide with one phosphorylated residue, on either RNMT T77 or S79 (Figure 1C). To discern the predominant site of phosphorylation, HeLa cells expressing HA-RNMT wild-type (WT), T77A, S79A, or T77A-S79A were incubated in ^{32}P orthophosphate, and label incorporation was determined (Figure 1D). Quantitation of two independent experiments indicated that the T77A-S79A mutation resulted in a 74% reduction in phosphorylation, whereas the T77A and S79A mutations resulted in 63% and 46% reduction in phosphorylation, respectively (Figure 1D). Therefore, the predominant site of RNMT phosphorylation in HeLa cells is T77. S79 may be phosphorylated less than T77 and/or may form part of the T77 kinase recognition site.

To characterize RNMT T77 phosphorylation, polyclonal antibodies were raised against RNMT 73CGKD**T**PSKKR82 phosphorylated on T77. HA-RNMT WT or T77A were immunoprecipitated from cell extracts via the HA tag, and western blots were performed to detect total RNMT and RNMT phosphorylated on T77 (pT77) (Figure 1E, left). RNMT was detected in HA-RNMT WT and T77A immunoprecipitates, whereas pT77 RNMT was only detected in HA-RNMT WT immunoprecipitates. Anti-pT77 antibodies did not detect RNMT immunoprecipitated in the absence of phosphatase inhibitors, consistent with recognition of wild-type RNMT only when phosphorylated (Figure 1E, right). Endogenous RNMT pT77 was also detected in anti-RNMT immunoprecipitates from HeLa cells (Figure 1F).

RNMT T77 Phosphorylation Is Reduced by CDK Inhibitors

RNMT T77 is embedded in a cyclin-dependent kinase (CDK) motif, T/S-P-x-K/R, adjacent to a cyclin recognition site, RxL (Dinkel et al., 2011; Figure 1G). Because CDK phosphorylates residues with proline at the +1 position, T77 could potentially be phosphorylated by this kinase family, whereas S79 could not (Songyang et al., 1994). We investigated whether CDK-cyclin complexes phosphorylate RNMT T77, focusing on cell cycle and transcription-associated kinases. RNMT T77 phosphorylation was assessed following treatment with roscovitine, which targets CDK1, CDK2, CDK5, CDK7, and CDK9, and RO-3306, which targets CDK1 and CDK2. Over a time course, RNMT T77 phosphorylation was first inhibited following incubation with 50 μM roscovitine for 10 min (Figure 1H). Across an inhibitor titration, inhibition of RNMT T77 phosphorylation was first observed with 20 μM roscovitine (Figure 1I). RNMT T77 phosphorylation was also reduced following a 5-min incubation with 9 μM RO-3306 (Figure 1J).

Because RNMT is recruited to transcribing RNA pol II, phosphorylation of RNMT T77 by the RNA pol II-associated CDKs was investigated. HeLa cells were incubated with flavopiridol and 5,6-dichloro-1-β-D-ribofuranosylbenzimidazole (DRB), inhibitors whose targets include the RNA pol II CTD kinases. Neither treatment inhibited RNMT T77 phosphorylation (Figures 1K and 1L).

RNMT T77 Is Phosphorylated during Late S and G2/M Phases

Use of roscovitine and RO-3306 implicated CDK1 or CDK2 in RNMT T77 phosphorylation, two kinases that drive cell cycle progression. CDK2 is predominantly active during S phase, and CDK1 during late S and G2/M phases. We investigated whether RNMT T77 is phosphorylated at a specific stage of the cell cycle, consistent with either CDK1 or CDK2-dependent phosphorylation. HeLa cells expressing HA-RNMT were synchronized at S phase entry by double thymidine block (Figure 2A, left) or synchronized at pro-metaphase by thymidine nocodazole block (Figure 2A, right). Cells were released into synchronous cell cycle progression for 12 hr. To confirm cell cycle progression, propidium iodide staining was used to determine the proportion of cells in each phase (Figure 2A, below blots), and extracts were analyzed for cyclin B1, E1/2, and A2 expression and histone H3 S10 phosphorylation, which oscillated as expected (Figure 2A). Total RNMT levels were relatively constant during the cell cycle, whereas RNMT T77 was phosphorylated in a cell cycle-dependent manner. Quantitation of T77 phosphorylation in three independent experiments demonstrated that, following double thymidine block, when cells were predominantly in S phase, pT77 RNMT was relatively low (Figure 2A, charts at the top). After 6 hr of progression through the cell cycle, pT77 RNMT elevated, corresponding with S phase

(F) Endogenous pT77 and total RNMT were detected in RNMT immunoprecipitates from HeLa cells.
(G) CDK and cyclin binding motifs in RNMT.
(H–L) HeLa cells expressing HA-RNMT were incubated with (H) 50 μM roscovitine, (I) a titration of roscovitine, (J) 9 μM RO-3306, (K) 1 μM flavopiridol, or (L) 50μM DRB for the times indicated. pT77 and total RNMT were detected in HA-RNMT immunoprecipitates.

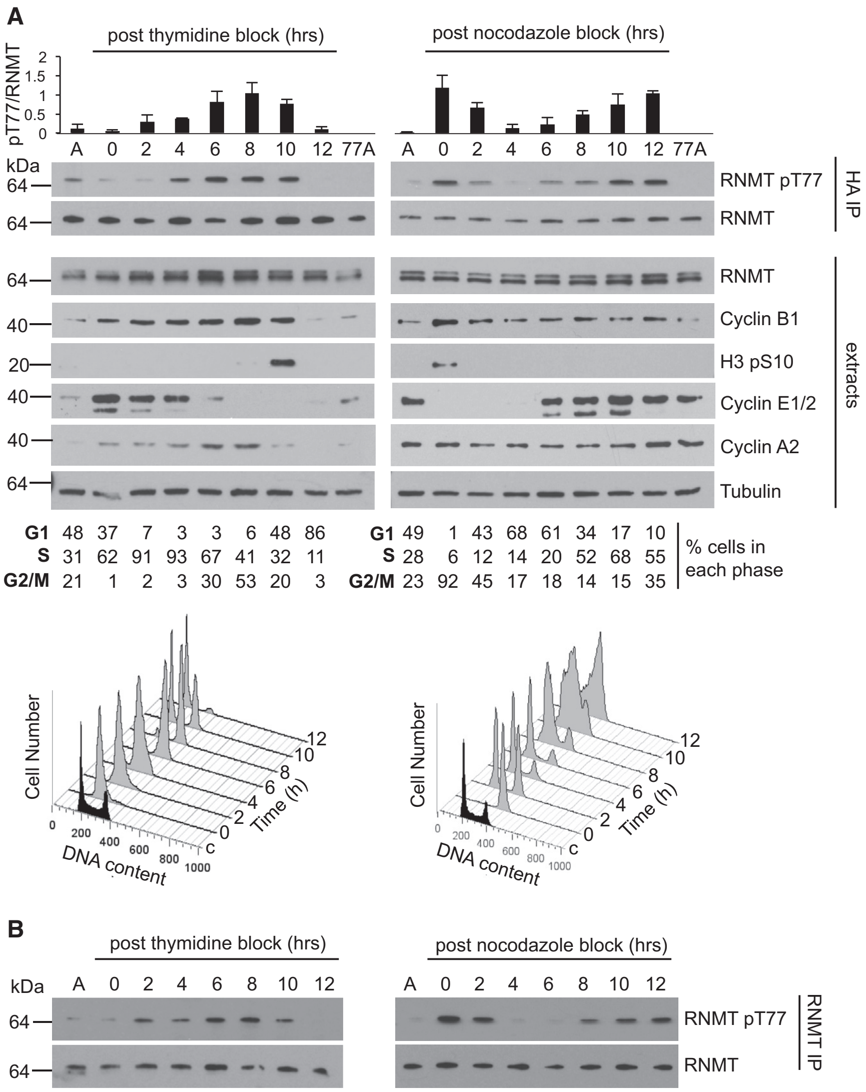

Figure 2. RNMT T77 Is Phosphorylated during G2/M Phase

(A) HeLa cells expressing HA-RNMT were synchronized by double thymidine or thymidine-nocodazole block and released as indicated. In these cells, asynchronous cells (A) and asynchronous cells expressing HA-RNMT T77A (77A), WBs were performed to detect pT77 and total RNMT in HA-RNMT immunoprecipitates and cell cycle markers indicated in cell extracts. Above the blots, a chart depicts the average pT77/RNMT signal and SD for three independent experiments, calculated from WB using ImageJ software. Flow cytometry cell cycle analysis and phase percentage are reported below the blots.

(B) HeLa cells were synchronized, and endogenous pT77 and total RNMT were detected in RNMT immunoprecipitates.

ending and entry into G2/M phase and was lost by 12 hr, corresponding with progression into G1 phase. Following thymidine nocodazole block, cells were predominantly in G2/M phase, and pT77 RNMT was elevated. On progression through G1 phase, pT77 RNMT decreased and remained low until 10-12 hr later, corresponding with the end of S phase and entry into G2/M phase. In summary, RNMT T77 is initially phosphorylated at the end of S phase and throughout G2/M phase, and phosphorylation is gradually lost during G1 phase. These findings were confirmed with endogenous RNMT (Figure 2B).

RNMT T77 Is Phosphorylated by CDK1-Cyclin B1

CDK1-cyclin B1 is the predominant cyclin-dependent kinase active during late S and G2/M phase, and therefore we investigated whether it is a RNMT T77 kinase. Expression of CDK1 and cyclin B1 was repressed in cells by siRNA transfection, with a G1 kinase, CDK3-cyclin E1, targeted as a control (Figure 3A). Transfection of CDK1 and cyclin B1 siRNA resulted in a significant reduction in pT77, whereas transfection of CDK3 and cyclin E1 siRNA resulted in a minor reduction. Analysis of cell extracts revealed that transfection of CDK and cyclin siRNAs inhibited expression of their direct target but also inhibited expression of each other, probably by indirect effects (Figure 3B). For example, CDK1 siRNA transfection resulted in a reduction in CDK1 and cyclin E1. However, transfection of CDK3 and cyclin E1 siRNA resulted in a similar reduction in cyclin E1 without a significant reduction in pT77 RNMT. Similarly, transfection of cyclin B1 siRNA resulted in a reduction in cyclin B1 and CDK3. However, a similar reduction in CDK3 was observed following transfection of CDK3 and cyclin E1 siRNA without significant loss of pT77 RNMT. This experiment provides evidence that CDK1-cyclin B1 is rate-limiting for pT77 phosphorylation but does not prove a direct effect.

We investigated whether CDK1-cyclin B1 could phosphorylate RNMT directly in vitro. Recombinant RNMT-RAM and RNMT T77A-RAM were incubated with CDK1-cyclin B1 and [γ-^{32}P] ATP and resolved by SDS-PAGE, and labeled bands were detected by phosphoimaging (Figure 3C). RNMT was phosphorylated by CDK1-cyclin B1 whereas RNMT T77A was not. RNMT was confirmed to be phosphorylated on T77 by western blot. Phosphorylation of RAM by CDK1-cyclin B1 was not detected (data not shown). To determine whether RNMT was phosphorylated by CDK1-cyclin B1 with the kinetics expected of an established substrate, the rates of phosphorylation of RNMT and histone H1, a CDK1-cyclin B1 substrate, were compared (Songyang et al., 1994; Figure 3D). In vitro phosphorylation reactions were performed over a time course using CDK1-cyclin B1 and a titration of recombinant RNMT-RAM or histone H1 (Figure S1). Reaction velocities were calculated for each substrate concentration, allowing K_M and k_{cat} determination

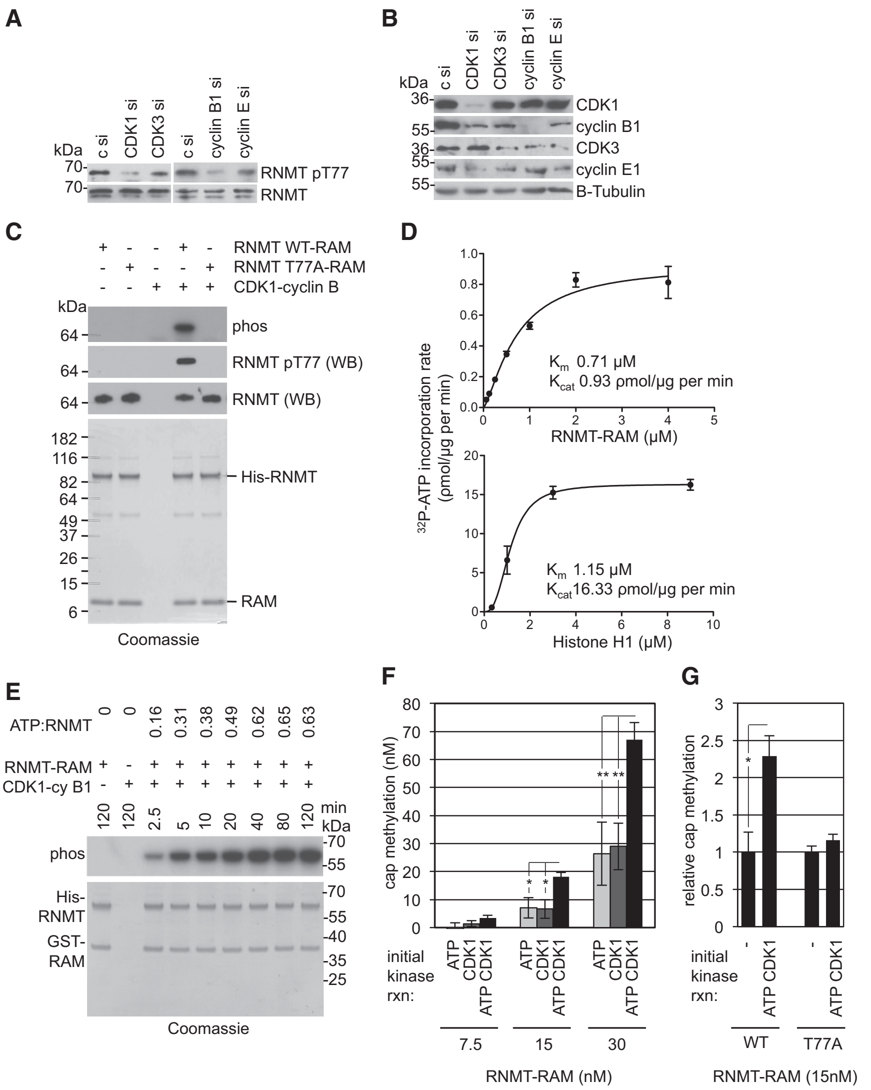

Figure 3. CDK1-Cyclin B1 Phosphorylates and Activates RNMT

HeLa cells were transfected with CDK1, CDK3, cyclin B1, and cyclin E1 siRNA.

(A and B) After 48 hr, (A) pT77 and total RNMT levels were analyzed in RNMT immunoprecipitates, and (B) CDK and cyclin levels were analyzed in cell extracts by WB. c, control.

(C) Recombinant His-RNMT (WT or T77A)-GST-RAM was incubated with activated CDK1-cyclin B1 and ^{32}P-ATP for 60 min and resolved by SDS-PAGE. Labeled bands were visualized by phosphoimaging. pT77 and total RNMT were visualized by WB and Coomassie stain.

(D) To assess enzyme kinetics, the CDK1-cyclin B1 kinase reaction was performed using a titration of RNMT or histone H1 over a time course (Figure S1). The charts depict reaction velocities for phosphorylation of RNMT-RAM or histone H1. Error bars represent SD for reaction velocity at each substrate concentration. k_{cat} and K_M values were calculated using an allosteric sigmoidal curve fit.

(E) As in (C), except a time course experiment was performed. Quantitation of the ATP:RNMT incorporation ratio is reported above.

(F) His-RNMT-GST-RAM was incubated with activated CDK1-cyclin B1 and ATP for 10 min as in (E) and then utilized in the cap methyltransferase assay. rxn, reaction.

(G) His-RNMT WT or 77A-GST-RAM were incubated in the presence and absence of ATP and CDK1-cyclin B1 for 10 min as above and then utilized in the cap methyltransferase assay.

Average and SD for three independent experiments is depicted. *$p < 0.05$ **$p < 0.005$, t test. See also Figure S1.

(Figure 3D). CDK1-cyclin B1 phosphorylated RNMT with a K_M of 0.71 μM and k_{cat} of 0.93 ρmol ATP/μg kinase/min and phosphorylated histone H1 with a K_M of 1.15μM and k_{cat} of 16.33 ρmol ATP/μg kinase/min. Therefore, CDK1-cyclin B1 phosphorylated RNMT-RAM with a similar K_M as histone H1. Although the rate of phosphorylation of histone H1 is higher than that of RNMT, histone H1 has multiple adjacent phosphorylation sites that facilitate rapid phosphorylation, whereas RNMT has one site (Godde and Ura, 2008).

RNMT T77 Phosphorylation Increases Cap Methyltransferase Activity

To determine whether T77 phosphorylation alters RNMT activity, recombinant RNMT-RAM was phosphorylated by CDK1-cyclin B1 prior to being analyzed for cap methyltransferase activity. Before measuring methyltransferase activity, it was important to determine the proportion of RNMT phosphorylated during the kinase assay. The kinase reaction was performed over a time course, and ^{32}P incorporation was quantitated (Figure 3E). In vitro kinase reactions are limited by substrate inhibition and kinase half-life. In this assay, saturation of ^{32}P incorporation was reached after 40 min, and maximal incorporation of 0.65 mol of phosphate to 1 mol of RNMT was achieved. Because only one CDK1-dependent phosphorylation site was detected on RNMT (T77) and mutation of this site abolished ^{32}P incorporation, it is likely that 65% RNMT was phosphorylated once. Although 65% RNMT is phosphorylated in vitro, this does not reflect the maximal phosphorylation in cells, which may be higher or lower. Following RNMT phosphorylation, the cap methyltransferase assay was performed (Figure 3F). Briefly, RNMT-RAM was incubated with a guanosine-capped transcript and the methyl donor, S-adenosyl methionine (SAM). Following the reaction, the substrate, G(5′)^{32}ppp(5′)G, and methylated product, m7G(5′)^{32}ppp(5′)G, were resolved by thin-layer chromatography (TLC) and quantitated by phosphoimaging. Phosphorylated RNMT was more than 2.5-fold more active than the unphosphorylated control (Figure 3F). These reactions were performed following a 10-min kinase reaction that resulted in approximately 38% RNMT phosphorylation. Although higher levels of phosphorylation could be achieved on longer incubation with CDK1-cyclin B1, prolonged incubation

in kinase buffer alone resulted in a significant reduction in RNMT cap methyltransferase activity (data not shown). To confirm that stimulation of RNMT activity was T77 phosphorylation-dependent, RNMT-RAM and RNMT T77A-RAM were incubated with and without CDK1-cyclin B1 and ATP, and the cap methyltransferase assay was performed (Figure 3G). As observed previously, RNMT-RAM activity was increased by incubation with CDK1-cyclin B1 and ATP but RNMT T77A-RAM activity was not.

KPNA2 RNMT Interaction Is Inhibited by RNMT T77 Phosphorylation

In addition to increasing cap methyltransferase activity, we investigated whether T77 phosphorylation influences other aspects of RNMT function. We investigated whether RNMT phosphorylation affects interaction with RAM. However, cellular RNMT WT and T77A were observed to interact with RAM equivalently, and their interaction did not alter during cell cycle progression, consistent with the observation that RAM binds to the RNMT catalytic domain (amino acids 120–476) (Figure S2; Gonatopoulos-Pournatzis et al., 2011). Because RNMT is recruited to transcription initiation sites via the N-terminal domain, we investigated whether this is influenced by T77 phosphorylation (Aregger and Cowling, 2013). Equivalent RNMT WT and T77A recruitment to transcription initiation sites was observed using a chromatin immunoprecipitation (IP) assay (Figure S3). Because T77 lies adjacent to a nuclear localization signal (80KKRK), we explored whether RNMT phosphorylation influences nuclear entry or retention. We visualized the nuclear accumulation of RNMT in asynchronous cells and found that RNMT WT, phospho-defective mutant T77A, and phospho-mimicking mutant T77D are predominantly nuclear (representative fields in Figures S4, S5, and S6). RNMT was observed to be excluded from chromatin during mitosis and rapidly re-enter the reformed nucleus following mitosis. The T77A and T77D mutations did not inhibit or enhance nuclear re-entry of RNMT (Figures S4, S5, and S6). In summary, we did not find evidence that T77 phosphorylation regulates RNMT interaction with RAM, recruitment to transcription initiation sites, or nuclear entry and retention.

To further characterize the function of T77 phosphorylation, we investigated the cellular proteins that interact with RNMT amino acids 57–92 using biotinylated peptides as affinity resins, unphosphorylated (T77) and phosphorylated on T77 (pT77) (Figure 4A). Peptides were incubated in cell extracts, and interacting proteins were purified via streptavidin pull-down. Proteins of approximately 150 and 90 kDa were observed to bind to the T77 peptide, and these interactions were reduced by T77 phosphorylation (Figure 4A). Several components of the nuclear protein import machinery were found to interact specifically with the unphosphorylated peptide. Three closely related proteins, Importin α-1 (KPNA2), Importin α-4 (KPNA3), and Importin α-3 (KPNA4) were identified by mass spectrometry in the 90-kDa band. Importin β-1 (KPNB1) was identified in the 150-kDa band. KPNA2 was the most abundant Importin α subunit identified in the T77 peptide pull-down and has been described previously to interact with RNMT (Shafer et al., 2005; Wen and Shatkin, 2000). Therefore, we investigated the relationship between RNMT T77 phosphorylation and the Importin α subunit, KPNA2. Cells were transfected with pcDNA5 Myc-tagged KPNA2 (Myc-KPNA2) or pcDNA5, and T77 and pT77 peptide pull-downs were performed on cell extracts. Myc-KPNA2 was observed to interact with the T77 but not the pT77 peptide (Figure 4B). In a similar assay, endogenous KPNA2 was also observed to interact with the T77 but not the pT77 peptide (Figure 4C). The interaction between full-length RNMT and KPNA2 was investigated. In extracts of cells expressing Myc-KPNA2, 9E10 (anti-Myc) antibody IP purified Myc-KPNA2 with RNMT, whereas a control (anti-HA) antibody was unable to immunoprecipitate either protein (Figure 4D). (The 9E10 antibody did not bind to RNMT directly [Figure 5A, lane 1]). Furthermore, anti-KPNA2 antibody IP purified endogenous KPNA2 with RNMT in cell extracts, whereas a matched control (anti-Tubulin) antibody did not immunoprecipitate either protein (Figure 4E). The interaction between RNMT and KPNA2 was confirmed to be direct using purified recombinant proteins. On glutathione affinity resin, recombinant RNMT was purified with glutathione S-transferase (GST)-KPNA2 but not GST (Figure 4F), and recombinant KPNA2 was purified with GST-RNMT but not GST (Figure 4G). When recombinant RAM was included in these studies, it did not bind to KPNA2 in the absence of RNMT (data not shown).

The influence of RNMT T77 phosphorylation on the interaction with KPNA2 was investigated. HeLa cell lines were created expressing HA-RNMT WT, T77A, T77D, and pcDNA5-Myc-KPNA2 and vector controls. RNMT T77D was included because, in certain contexts, aspartic acid can mimic the behavior of phosphorylated threonine. 9E10 antibody was used to immunoprecipitate Myc-KPNA2 with endogenous RNMT and HA-RNMT (lanes 2–4, Figure 5A). Significantly more HA-RNMT T77A co-purified with Myc-KPNA2 than either WT or T77D, despite being expressed equivalently, consistent with cellular HA-RNMT WT being phosphorylated, which reduces the interaction with KPNA2. The fact that the T77D mutation also reduces the interaction of RNMT with KPNA2 suggests that this mutation functions as a "phospho-mimic." Similar results were gained addressing the interaction of HA-RNMT with endogenous KPNA2 (Figure 5B). Anti-KPNA2 antibodies were used to immunoprecipitate KPNA2. The levels of HA-RNMT T77A immunoprecipitated with KPNA2 were significantly higher than those of HA-RNMT T77D and slightly higher than those of HA-RNMT WT.

Because T77 phosphorylation inhibits the interaction of RNMT with KPNA2, the interaction of these proteins is predicted to be low at G2/M phase when RNMT T77 phosphorylation is high. Cells were synchronized by double thymidine block. 2 hr (S phase) and 8 hr (G2/M phase) following release, cell extracts were subject to immunoprecipitation (IP) with 9E10 antibody to purify Myc-KPNA2 (Figure 5C) and with anti-KPNA2 antibody to purify the endogenous protein (Figure 5D). As predicted, more RNMT was immunoprecipitated with KPNA2 during S phase than in G2/M phase. Treatment of cells with RO-3306 or roscovitine, which inhibit RNMT T77 phosphorylation, increased the interaction of RNMT with endogenous and Myc-tagged KPNA2 (Figures 5C and 5D).

KPNA2 Inhibits RNMT Activity

RNMT T77 phosphorylation increases cap methyltransferase activity in vitro in the absence of KPNA2 (Figure 3). Because RNMT

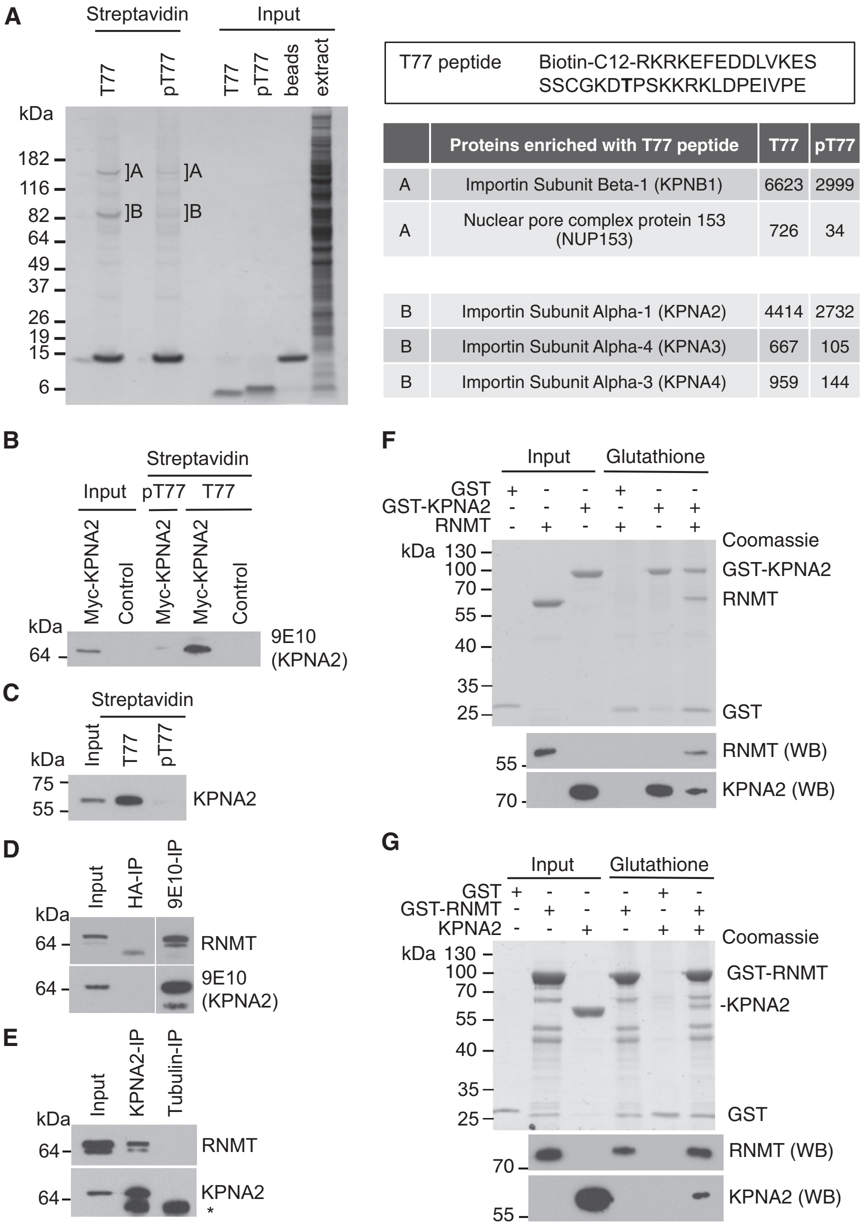

	Proteins enriched with T77 peptide	T77	pT77
A	Importin Subunit Beta-1 (KPNB1)	6623	2999
A	Nuclear pore complex protein 153 (NUP153)	726	34
B	Importin Subunit Alpha-1 (KPNA2)	4414	2732
B	Importin Subunit Alpha-4 (KPNA3)	667	105
B	Importin Subunit Alpha-3 (KPNA4)	959	144

Figure 4. RNMT T77 Phosphorylation Inhibits Interaction with KPNA2

(A) HeLa cell extracts were incubated with biotinylated-RNMT 73CGKD(**T**)PSKKR82 peptide, either T77 unphosphorylated (T77) or phosphorylated (pT77). Peptides and associated proteins were affinity-purified, resolved by SDS-PAGE, and Coomassie blue-stained. Proteins in A and B were identified by mass spectrometry. Mascot scores are reported.

(B) HeLa cells were transfected with pcDNA5 Myc-KPNA2 or vector control, and proteins were affinity-purified from cell extracts on RNMT peptides. Myc-KPNA2 was detected by WB performed using 9E10 antibody.

(C) HeLa cell extracts were affinity-purified on RNMT peptides, and endogenous KPNA2 was detected by WB.

(D) HeLa cells were transfected with pcDNA5 Myc-KPNA2. 9E10 antibodies were used to immunoprecipitate Myc-KPNA2 and anti-HA antibodies used as a control. WBs were performed to detect Myc-KPNA2 and RNMT (adjacent panels are components of the same blot).

(E) Anti-KPNA2 antibody was used to immunoprecipitate KPNA2 from cell extracts, with anti-Tubulin antibody used as a control. WBs were performed to detect KPNA2 and RNMT. The star indicates a non-specific band.

(F) Recombinant GST or GST-KPNA2 was incubated with recombinant (untagged) RNMT and affinity-purified on glutathione-Sepharose. Inputs and proteins eluted were resolved by SDS-PAGE and Coomassie blue-stained or analyzed by WB to detect RNMT and KPNA2.

(G) As (F), except GST-RNMT was incubated with purified recombinant KPNA2.

See also Figures S2 and S3.

T77 phosphorylation regulates interaction with KPNA2, it was relevant to determine the influence of KPNA2 on RNMT activity. An in vitro cap methyltransferase assay was performed on recombinant RNMT and a titration of KPNA2. KPNA2 inhibited cap methyltransferase activity in a dose-dependent manner (Figure 5E). A previous publication determined the KPNA2 domains that interact with RNA (amino acids 1–72) and RNMT (amino acids 455–529) (Wen and Shatkin, 2000). Using GST-KPNA2 pull-down, we confirmed that KPNA2 1–455 does not interact with RNMT whereas KPNA2 72–529 does (Figure 5F). The reciprocal interactions were also observed (Figure S7) and confirmed in cell extracts (Figure 5G). The RNMT-binding mutant KPNA2 1–455 did not inhibit RNMT-RAM cap methyltransferase activity (Figure 5H). The RNA-binding mutant KPNA2 72–529 also did not inhibit RNMT activity, suggesting that KPNA2 requires both domains to inhibit RNMT-RAM activity (Figure 5H). Unfortunately it was not possible to cleanly investigate the contribution of KPNA2 to RNMT activity in cells because inhibition of KPNA2 results in loss of nuclear RNMT.

In summary, RNMT T77 phosphorylation increases the activity of RNMT directly and indirectly by preventing the repressive action of KPNA2. The relative contribution of these two mechanisms to cellular RNMT may vary depending on the cell lineage and physiological context.

RNMT T77 Phosphorylation Correlates with Cellular Cap Methyltransferase Activity

We had observed that RNMT T77 phosphorylation is elevated at the beginning of G1 phase and falls as the cell cycle progresses (Figure 2) and that RNMT T77 phosphorylation directly and indirectly activates RNMT (Figures 3, 4, and 5). A prediction from these observations is that cellular cap methyltransferase activity is elevated at the initiation of G1 phase and falls during the cell

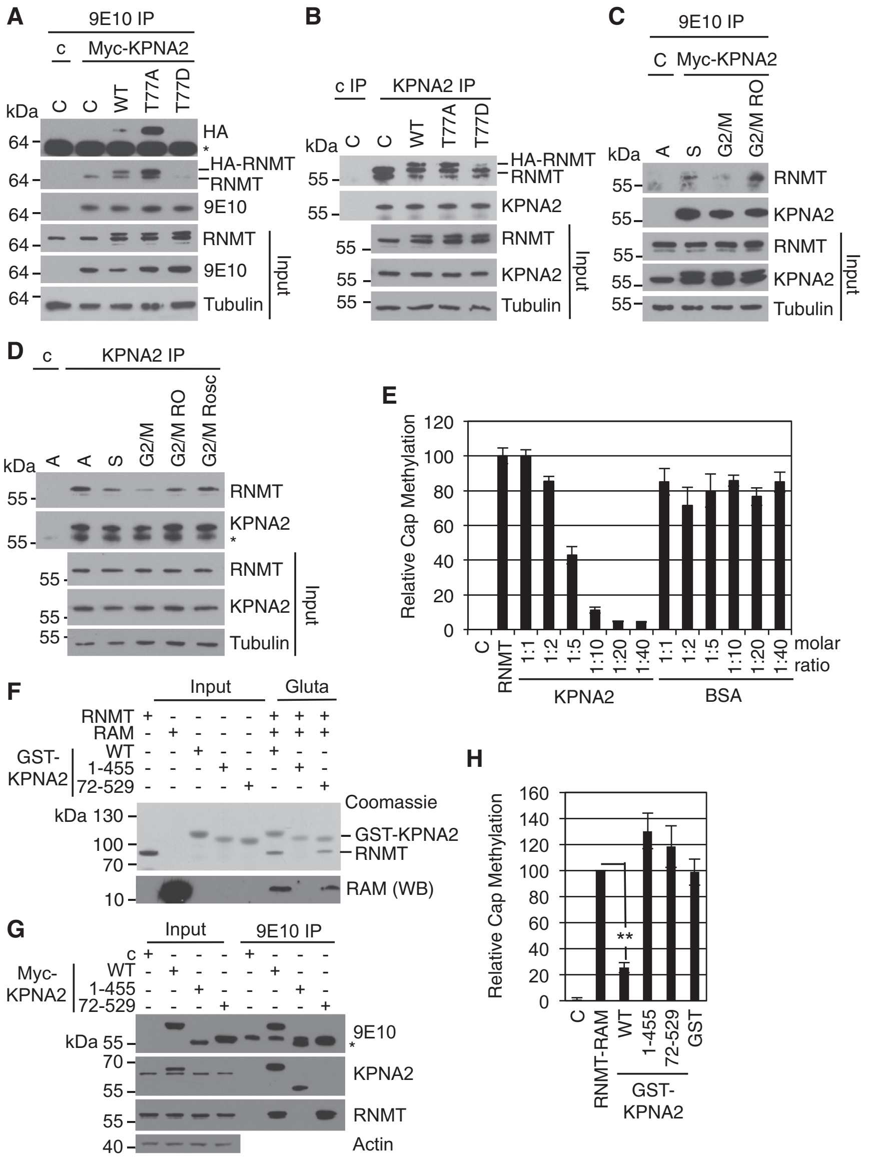

Figure 5. RNMT Phosphorylation Reduces Inhibition by KPNA2

(A) HeLa cells expressing HA-RNMT WT, T77A, T77D, or vector control (C) were transfected with pcDNA5 Myc-KPNA2 or vector control (C). 9E10 antibody was used to immunoprecipitate Myc-KPNA2, and WBs were performed to detect HA-RNMT, RNMT, and Myc-KPNA2.

(B) KPNA2 IP was performed on HeLa cells expressing HA-RNMT WT, T77A, T77D, or a vector control using anti-KPNA2 antibody, and KPNA2 and RNMT were detected by WB.

(C) HeLa cells were transfected with pcDNA5 Myc-KPNA2. Cells were released from double thymidine block for 2 hr (S) or 8 hr (G2/M), including incubation with 9 μM RO-3306 for 15 min (G2/M RO). Asynchronous cells transfected with the vector control were used as a control. 9E10 antibody was used to immunoprecipitate Myc-KPNA2, and WBs were performed to detect RNMT and KPNA2.

(D) HeLa cells were treated as in (C), except that G2/M cells were also treated with 50 μM roscovitine (G2/M Rosc) for 15 min. Anti-KPNA2 antibodies were used to immunoprecipitate KPNA2 from cell extracts using anti-Tubulin antibodies as a control. WBs were performed to detect KPNA2, RNMT, and Tubulin in extracts and immunoprecipitates.

(E) A cap methyltransferase assay was performed using 40 nM recombinant RNMT and titration of recombinant KPNA2 or BSA. Activity is reported relative to the RNMT control.

(F) Recombinant RNMT and RAM were incubated with recombinant GST-KPNA2 WT, 1–455, 72–529, or GST alone, and complexes were affinity-purified with glutathione-Sepharose. Inputs and eluates were resolved by SDS-PAGE and Coomassie blue-stained, and RAM was detected by WB.

(G) HeLa cells were transfected with pcDNA5 Myc-KPNA2 WT, 1–455, 72–529, or vector control (C). 9E10 antibody was used to immunoprecipitate Myc-KPNA2, and WB was performed to detect Myc-KPNA2, KPNA2, RNMT, and Tubulin. (Note that the KPNA2 antibody raised against the N terminus does not recognize KPNA2 72–529.)

(H) A cap methyltransferase assay was performed using recombinant RNMT-RAM and recombinant KPNA2 WT, 1–455, 72–529, or GST alone. The charts depict the average and SD of four experiments. **$p < 0.005$, t test. Nonspecific bands are indicated with a star.

See also Figures S4, S5, S6, and S7.

cycle, in correlation with RNMT T77 phosphorylation. To investigate the temporal nature of RNMT activity, cells were synchronized at prometaphase by thymidine-nocodazole block and released into G1 phase. At hourly intervals, nuclear extracts were prepared in which RNMT pT77 was determined by western blot and cap methyltransferase activity by in vitro assay (Figure 6A). At the initiation of G1 phase, RNMT pT77 levels were relatively high and remained so until 3 hr into G1 phase. Cap methyltransferase activity in the same extracts was also relatively high at the beginning of G1 phase and remained so until 3 hr later, when activity fell 2-fold. To further probe the relationship between RNMT T77 phosphorylation and cellular cap methyltransferase activity during G1 phase, cells were treated with the kinase inhibitor roscovitine for 30 min prior to lysis. Roscovitine inhibited RNMT T77 phosphorylation detected 1 hr into G1 phase (Figure 6B). As observed previously, cap methyltransferase activity was relatively high 1 hr after the initiation of G1 phase, and treatment with roscovitine inhibited this activity (Figure 6B). Therefore, cellular cap methyltransferase activity correlates with high RNMT T77 phosphorylation at the beginning of G1 phase.

RNMT T77 Phosphorylation and Gene Expression

The effect of RNMT T77 phosphorylation on gene expression was examined. HA-RNMT WT and T77A were expressed in HeLa cells for 24 hr to focus on the direct rather than indirect

effects of inhibiting RNMT phosphorylation. Because the mRNA cap can influence transcript expression, stability, and splicing, extensive RNA sequencing analysis was performed. However, no significant difference in transcript expression or splicing was found in response to expression of RNMT T77A (data not shown). The mRNA cap is critical for eIF4E binding and translation initiation. Therefore, we performed a proteomic analysis of three independent HeLa whole-cell preparations expressing RNMT WT or T77A. 4,906 proteins were identified (Table S1), 1,910 of which were detected in three independent experiments, therefore qualifying for statistical analysis. 144 genes were differentially expressed in response to RNMT T77A (Table S2): 53 repressed in response to expression of RNMT 77A and 91 activated (t test p values < 0.05; Figure 6C; Table S2). Gene ontology (GO) analysis for biological processes indicated a broad effect of inhibiting RNMT T77 phosphorylation, including genes involved in the cell cycle, apoptosis, metabolism, and RNA processing (Table S3). GO analysis was plotted using the REVIGO suite to reduce redundancy (Figure 6D; Supek et al., 2011).

To validate the proteomic results, the expression of four genes, BOP1, TOMM70A, DDX18, and Skp2, was analyzed (Table S2). In response to RNMT T77A expression, mass spectrometry analysis revealed that BOP1, TOMM70A, DDX18, and Skp2 expression was significantly repressed whereas β-Tubulin was not (Figure 6E, charts). Western blot analysis confirmed these results (Figure 6E). Although the sequencing analysis found that these genes were not regulated at the transcript level, expression of RNMT T77A did reduce the proportion of BOP1, TOMM70A, and Skp2 transcripts with an m7G cap, consistent with a reduction of their translation and protein expression (Figure 6F). The m7G level was determined by m7G IP followed by gene-specific RTPCR. RNMT T77A expression did not reduce the proportion of β-Tubulin transcripts with an m7G cap or reduce the protein level (Figures 6E and 6F). Unfortunately not all transcripts are amenable to m7G IP, including DDX18, possibly because of RNA secondary structure affecting anti-m7G antibody access to the cap.

RNMT T77 Phosphorylation and Cell Proliferation

To determine the biological effect of T77 phosphorylation, the proliferation rate of cells expressing RNMT T77A was examined. HeLa cells were depleted of RNMT-RAM by transfection of RAM siRNA (which depletes RNMT-RAM more efficiently than RNMT siRNA). This resulted in a reduction in RNMT and RAM expression and a reduction in cell proliferation (Figures 7A and 7B). HA-RNMT WT and T77A were expressed in these cells with FLAG-RAM, resulting in equivalent expression (Figure 7A). HA-RNMT WT but not T77A rescued the proliferation defect (Figure 7B). The effect of the T77A mutation on the ability of RNMT to induce anchorage-independent proliferation of mammary epithelial cells was also investigated. For this assay, RNMT-GFP fusions were used because GFP increases RNMT expression and colony formation (Aregger and Cowling, 2013; Cowling, 2010). As observed previously, expression of RNMT-GFP results in over 20% of mammary epithelial cells exhibiting a transformed phenotype, forming colonies in soft agar, whereas expression of RNMT T77A-GFP and GFP alone resulted in approximately 5% of cells forming colonies. Therefore, in both assays, the T77A mutation causes a defect in proliferation. In conclusion, RNMT T77 phosphorylation is required for biologically significant gene expression and cell proliferation.

DISCUSSION

The mRNA cap is a structure critical for gene expression, recruiting complexes that mediate transcript processing and translation initiation. We report that the mRNA cap N-7 methyltransferase enzymic subunit RNMT, which completes basic cap formation, is activated by phosphorylation during G2/M phase of the cell cycle. When transcription reinitiates after mitosis, enhanced RNMT activity increases the rate of capping essential to make functional mRNA.

CDK1 Activates RNMT

The predominant site of RNMT phosphorylation is amino acid T77, which lies within the regulatory domain of the enzyme (Aregger and Cowling, 2013). RNMT T77 phosphorylation increases catalytic activity and reduces an inhibitory interaction with KPNA2. RNMT T77 is embedded in a CDK recognition motif adjacent to a cyclin recognition motif and is phosphorylated during late G2/M phase until mid-G1 phase. The predominant kinase operational during this phase is CDK1-cyclin B1, which phosphorylates RNMT T77 directly. Other CDK-cyclin complexes are operational at this phase and may also phosphorylate RNMT. As a consequence of RNMT phosphorylation, cellular mRNA cap methyltransferase activity is relatively high during the first few hours of G1 phase and then drops. Inhibition of RNMT T77 phosphorylation by use of a threonine-to-alanine substitution results in a reduction in protein synthesis and cell proliferation.

Because mRNA cap methylation can influence transcript abundance, processing, and translation initiation we investigated the response of the proteome to inhibiting RNMT T77 phosphorylation. The genes most dependent on RNMT T77 phosphorylation extend across many biological processes. From our analysis, no particular gene family or gene sequence/arrangement could be identified that favored sensitivity to RNMT phosphorylation. It may be that a combination of chromatin context, transcriptional speed, untranslated region sequence/structure, and other factors contribute to sensitivity to RNMT phosphorylation. We performed RNA sequencing analysis to investigate the effect of inhibiting RNMT phosphorylation on transcript abundance and splicing (data not shown). Despite adequate depth of sequencing, we found no significant effect on transcript abundance or splicing, suggesting that the predominant effect of the methylation of the mRNA cap in HeLa cells is translation initiation.

Function of Cell Cycle-Dependent mRNA Cap Methylation

During mitosis, the nuclear membrane breaks down, allowing complete division of the cellular contents. At this time, RNA pol II and most of its associated factors detach from chromatin, with only a few bookmarks of transcription initiation remaining (Zaidi et al., 2010). When the nuclear membrane is reformed at the end of M phase, RNA pol II is rapidly recruited to promoters, accompanied by a burst of transcription and subsequent

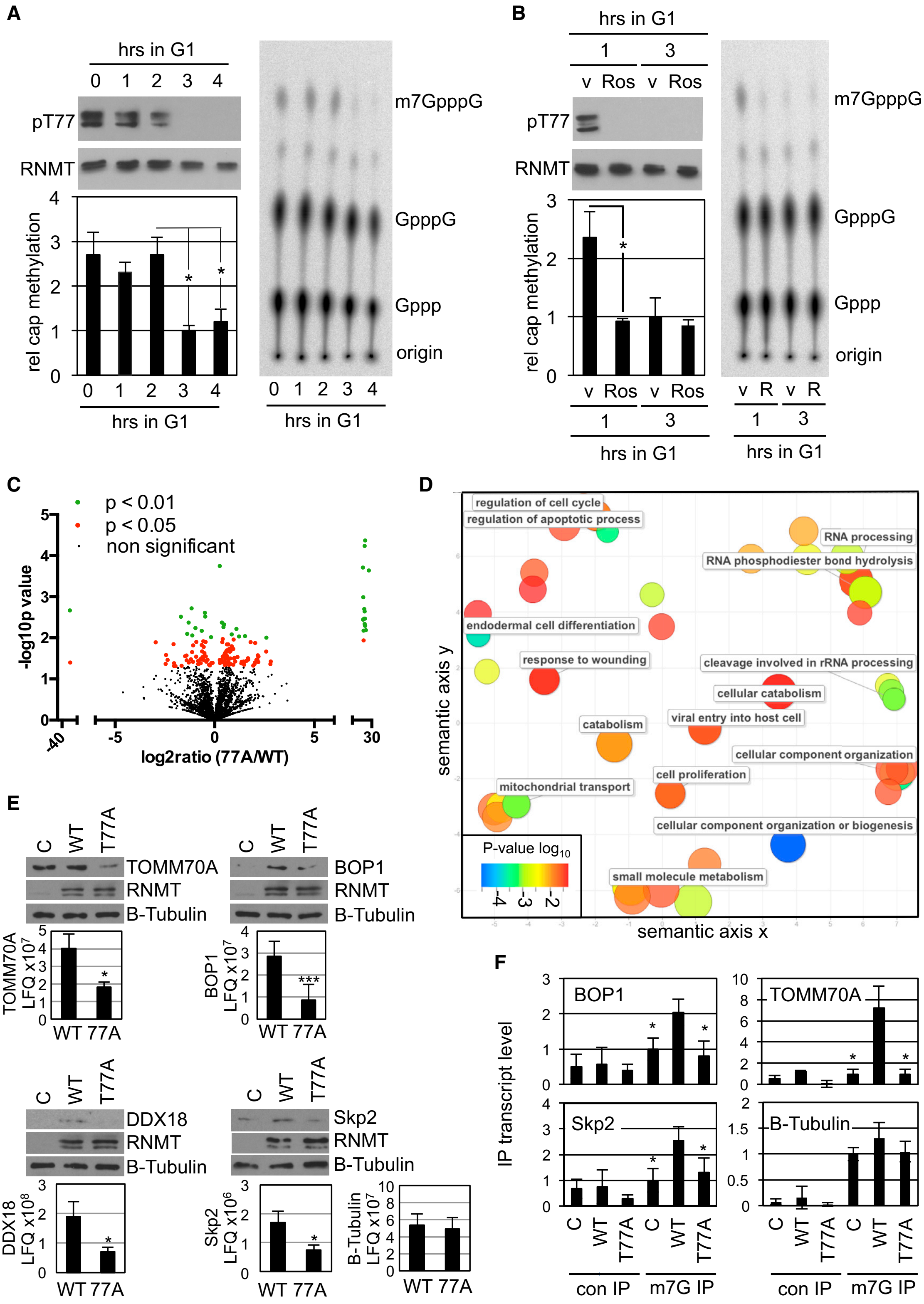

Figure 6. RNMT Phosphorylation Is Required for Gene Expression

(A) HeLa cells synchronized by thymidine-nocodazole block were released into G1 phase for the hours indicated. pT77 and total RNMT were detected in RNMT immunoprecipitates by WB. Relative cap methyltransferase activity of the same cell extracts was determined. A sample result is presented,

(legend continued on next page)

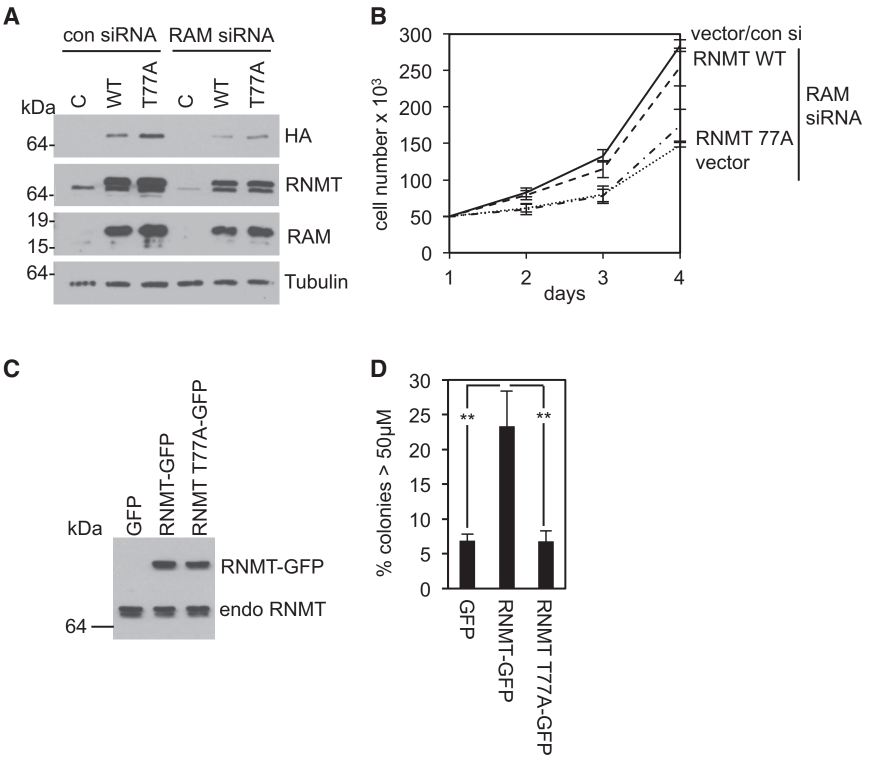

Figure 7. RNMT T77 Phosphorylation Is Required for Cell Proliferation

(A) HeLa cells were transfected with control or RAM siRNA, plus pcDNA5, pcDNA5 HA-RNMT WT, or T77A and pcDNA5 FLAG-RAM. RNMT and RAM were detected by WB.

(B) The chart depicts average cell number and SD for three independent experiments.

(C) Immortalized mammary epithelial cells (IMECs) expressing pcDNA GFP, RNMT-GFP or RNMT T77A-GFP were analyzed by WB for expression of endogenous RNMT and RNMT-GFP.

(D) IMEC lines were plated in suspension in soft agar. The number of established colonies (over 50 μM in diameter) after 7 days is reported. The chart depicts the average and SD of three experiments. $^{**}p < 0.005$, t test.

translation (Dynlacht, 1997; Pyronnet and Sonenberg, 2001). CDK1-dependent phosphorylation of RNMT increases cap methyltransferase activity at the initiation of G1 phase, coordinating the manufacture of functional transcripts with the transcriptional burst. Not only does T77 phosphorylation increase cap methyltransferase activity, it also inhibits the negative effect of KPNA2 on RNMT activity. This is particularly important after completion of mitosis when the cellular pool of RNMT is being imported into the nucleus by interaction with KPNA2 (Shafer et al., 2005).

Although RNMT T77 phosphorylation occurs in a cell cycle-dependent manner, its effects are independent of cell cycle phase. Expression of RNMT T77A inhibits expression of a subset of proteins, but these are not synthesized at a specific cell cycle phase or check point (Stumpf et al., 2013; Tanenbaum et al., 2015). Furthermore, although preventing RNMT phosphorylation inhibits cell proliferation, the distribution of cells through the cell cycle phases is unaffected (data not shown). These observations may appear contradictory. However, they are perhaps unsurprising given that most mammalian gene promoters contain promoter-proximal paused RNA pol II, and, therefore, capping can be uncoupled from, and potentially occurs much prior to, productive elongation (Buratowski, 2009; Nilson et al., 2015; Perales and Bentley, 2009). As a result, loss of RNMT phosphorylation reduces gene expression and cell proliferation independently of cell cycle stage.

Regulation of mRNA Cap Methylation

The catalytic domains of mRNA cap methyltransferases are conserved in sequence and structure in eukaryotes (Fabrega et al., 2004). In contrast, eukaryotes vary significantly in the sequence and length of the cap methyltransferase N-terminal regulatory domain. The RNMT N-terminal domain has some conservation with Vaccinia virus cap methyltransferase N-terminal extension, which folds back over the SAM binding site, occluding the ligand (De la Peña et al., 2007). If RNMT has a

indicating migration of substrate, GpppG, and product, m7GpppG, on TLC. Average and SD are presented in the chart for three biologically independent experiments.

(B) As in (A), except cells were treated with 50 μM roscovitine for 30 min prior to analyses. v, vector.

(C) Label-free proteomic analysis of HeLa cells expressing HA-RNMT WT and T77A was performed on three independent samples. Data were analyzed by Maxquant for significance, and identified proteins are represented by volcano plot depicting the average expression ratio in cells expressing HA-RNMT 77A versus WT.

(D) REVIGO rationalization of GO term analysis of proteins repressed in cells expressing RNMT T77A in comparison with WT. Axes represent semantic space used to group GO terms of related biological processes. The color and size of each bubble indicates the p value for each GO term versus the human proteome. The bubble size increases and color changes from red to blue with increasing p value. The proximity of the bubbles reflects the relatedness of GO terms.

(E) BOP-1, TOMM70A, DDX18, and Skp2 were detected by WB in extracts of cells expressing HA-RNMT WT, T77A, and vector control. For three independent preparations of cells expressing HA-RNMT WT and 77A, the charts indicate the average label-free quantitation (LFQ) and SD of proteins detected in the mass spectrometry analysis.

(F) RNA was immunoprecipitated from the same cells using an anti-m7G antibody or control, and RT-PCR was used to detect m7G-capped transcripts relative to total transcripts. $^{*}p > 0.05$, $^{**}p > 0.001$, t test.

See also Tables S1, S2, and S3.

similar structure, then post-translational modifications such as T77 phosphorylation may alter the conformation of this N-terminal domain, therefore altering accessibility of the SAM binding domain. We previously established that the RNMT N-regulatory domain influences the catalytic activity of RNMT and its promoter recruitment (Aregger and Cowling, 2013). In addition to T77 phosphorylation, we observed that the regulatory domain is a region of significant post-translational modifications, including phosphorylation, methylation, and acetylation, whereas the catalytic domain is particularly deficient in modification (data not shown). It seems likely that other modifications in addition to T77 phosphorylation will have global or gene-specific effects on proteome, providing a route for other extra- or intracellular signaling pathways to coordinately regulate RNA regulons and cellular function.

EXPERIMENTAL PROCEDURES

Mass Spectrometry Analysis

50 mg cell extract was precleared for 5 hr with 50 μl murine IgG-conjugated agarose (Sigma) and 20 μl protein A/G agarose (Santa Cruz Biotechnology). HA-RNMT was immunoprecipitated overnight using 20 μl mouse monoclonal anti-HA antibody-conjugated agarose (Sigma). Immunoprecipitates were resolved SDS-PAGE and Brilliant Blue G-Colloidal-stained (Sigma). After trypsin digestion, samples were analyzed by LC-MS/MS on a 4000 QTrap mass spectrometer for protein identification, providing 66% coverage of human RNMT. Precursor ion scanning followed by LC-MS/MS performed on an LTQ Orbitrap Velos Pro mass spectrometer was used to detect phosphopeptides. The LC-MS/MS data were used to interrogate the SwissProt or International Protein Index (IPI) human databases using the Mascot Daemon to identify site(s) of phosphorylation.

Cell Cycle Synchronization

For the double thymidine block, 1×10^6 cells were seeded on 10-cm plates. 2.5 mM thymidine was added for 16–18 hr. Cells were washed with PBS and released into growth medium for 8 hr. Cells were cultured in 2.5 mM thymidine again for 18 hr, washed, and released into growth medium.

For the thymidine-nocodazole block, 2×10^6 cells were seeded on 10-cm plates. 2.5 mM thymidine was added for 24 hr. Cells were washed and released into growth medium for 3 hr. 100 ng/ml nocodazole was added for 13 hr. Cells were washed and released into growth medium.

In Vitro Phosphorylation

0.33 μM His-RNMT (WT or T77A)-GST-RAM 1–90, 0.01 μmol CDK1-Cyclin B1 (New England Biolabs), 100 μM ATP (cold or [γ-^{32}P] ATP [500 cpm/pmol]) (PerkinElmer), 10 mM $MgCl_2$, 2 mM DTT, 50 mM Tris (pH 7.5), and 0.1 mM EGTA were incubated in 30 μl at 30°C while shaking at 1,200 rpm. Following SDS-PAGE, RNMT bands were excised and analyzed by scintillation counter to estimate the stoichiometry of ^{32}P incorporation into RNMT.

Enzyme kinetics were measured using 0.01 μM CDK1-Cyclin B1 and a titration of His-RNMT GST-RAM 1–90 or histone H1 using the previous conditions. Phosphorylated protein was quantified following SDS-PAGE and autoradiography by image densitometry. Slopes calculated from linear regression obtained in Figure S1 were plotted as the reaction velocities in Figure 3D.

In Vitro Phosphorylation Coupled to Methyltransferase Activity Assay

In vitro phosphorylation was performed for 10 min as above, and the reaction was diluted in methyltransferase (MT) buffer and subjected to cap methyltransferase assay. 330 nM RNMT-RAM was present in the kinase assay, which was diluted to a maximum of 30 nM in the cap methyltransferase assay.

Cap Methylation Assay

The RNA substrate was a 55-nt transcript transcribed from linearized pGEM CE4 using T7 (Promega). 200 ng transcript was capped with 0.1 μg RNGTT, 10 μCi [α-^{32}P]GTP (PerkinElmer), and 40 U RNasin. For the cap methylation assay, recombinant RNMT was incubated with 2 ng substrate, 2 μM S-adenosyl methionine, and 10 U RNasin at 30°C for 10 min. Transcripts were purified and digested with 0.75 U P1 nuclease (Sigma) for 60 min at 37°C to release free GpppG and m7GpppG. Cap structures were resolved by 0.4 M ammonium sulfate on polyethyleneimine (PEI) cellulose plates. Labeled GpppG and m7GpppG spots were visualized and quantified by phosphoimager.

Cap Methylation Assay Performed on Cell Extracts

10^6 HeLa cells were plated on a 10-cm dish. The next day, a thymidine-nocodazole block was performed. Following release into G1 phase, each hour cells were lysed in Dignam A buffer, and nuclear proteins were extracted using Dignam C buffer (Dignam et al., 1983). The buffers contained 10 μM leupeptin, 1 μM pepstatin, 10 μg/ml aprotinin, 1 mM DTT, and 1:1,000 phosphatase inhibitor cocktail 2 and 3 (all from Sigma). The assay was performed on 3 μg nuclear extract, as above.

Cell culture, flow cytometry, immunological methods, and protein and RNA analyses are described in the Supplemental Experimental Procedures.

SUPPLEMENTAL INFORMATION

Supplemental Information includes Supplemental Experimental Procedures, seven figures, and three tables and can be found with this article online at http://dx.doi.org/10.1016/j.molcel.2016.02.008.

AUTHOR CONTRIBUTIONS

M.A., A.K., D.V., M.E.F.-S., F.A.I.-V., S.W., and V.H.C. designed and performed the experiments and wrote the paper.

ACKNOWLEDGMENTS

Research was funded by a Medical Research Council (MRC) senior non-clinical fellowship and Lister Prize research fellowship (to V.H.C.), a Cancer Research UK Ph.D. studentship (to M.A.), Wellcome Trust Strategic Award 083524/Z/07/Z, and Centre Award 097945/Z/11/Z. We thank the V.H.C. and Alessi labs, Sandra Moser and Saskia Hutten, the Dundee Imaging Facility (Wellcome Trust Technology Platform Award 097945/B/11/Z and MRC Next Generation Optical Microscopy Award MR/K015869/1), FingerPrints Proteomics, Flow Cytometry and Karim Labib, Julian Blow, and Leah Torrie for assistance and discussions. We thank the Division of Signal Transduction Therapy funded by AstraZeneca, Boehringer-Ingelheim, GlaxoSmithKline, Janessen, Merck-Serono, and Pfizer.

Received: September 4, 2015
Revised: December 11, 2015
Accepted: February 5, 2016
Published: March 3, 2016

REFERENCES

Aregger, M., and Cowling, V.H. (2012). E2F1-dependent methyl cap formation requires RNA pol II phosphorylation. Cell Cycle *11*, 2146–2148.

Aregger, M., and Cowling, V.H. (2013). Human cap methyltransferase (RNMT) N-terminal non-catalytic domain mediates recruitment to transcription initiation sites. Biochem. J. *455*, 67–73.

Buratowski, S. (2009). Progression through the RNA polymerase II CTD cycle. Mol. Cell *36*, 541–546.

Cole, M.D., and Cowling, V.H. (2009). Specific regulation of mRNA cap methylation by the c-Myc and E2F1 transcription factors. Oncogene *28*, 1169–1175.

Cowling, V.H. (2010). Enhanced mRNA cap methylation increases cyclin D1 expression and promotes cell transformation. Oncogene *29*, 930–936.

Cowling, V.H., and Cole, M.D. (2007). The Myc transactivation domain promotes global phosphorylation of the RNA polymerase II carboxy-terminal domain independently of direct DNA binding. Mol. Cell. Biol. *27*, 2059–2073.

De la Peña, M., Kyrieleis, O.J., and Cusack, S. (2007). Structural insights into the mechanism and evolution of the vaccinia virus mRNA cap N7 methyl-transferase. EMBO J. *26*, 4913–4925.

Dignam, J.D., Lebovitz, R.M., and Roeder, R.G. (1983). Accurate transcription initiation by RNA polymerase II in a soluble extract from isolated mammalian nuclei. Nucleic Acids Res. *11*, 1475–1489.

Dinkel, H., Chica, C., Via, A., Gould, C.M., Jensen, L.J., Gibson, T.J., and Diella, F. (2011). Phospho.ELM: a database of phosphorylation sites–update 2011. Nucleic Acids Res. *39*, D261–D267.

Dynlacht, B.D. (1997). Regulation of transcription by proteins that control the cell cycle. Nature *389*, 149–152.

Fabrega, C., Hausmann, S., Shen, V., Shuman, S., and Lima, C.D. (2004). Structure and mechanism of mRNA cap (guanine-N7) methyltransferase. Mol. Cell *13*, 77–89.

Fernandez-Sanchez, M.E., Gonatopoulos-Pournatzis, T., Preston, G., Lawlor, M.A., and Cowling, V.H. (2009). S-adenosyl homocysteine hydrolase is required for Myc-induced mRNA cap methylation, protein synthesis, and cell proliferation. Mol. Cell. Biol. *29*, 6182–6191.

Ghosh, A., Shuman, S., and Lima, C.D. (2011). Structural insights to how mammalian capping enzyme reads the CTD code. Mol. Cell *43*, 299–310.

Godde, J.S., and Ura, K. (2008). Cracking the enigmatic linker histone code. J. Biochem. *143*, 287–293.

Gonatopoulos-Pournatzis, T., and Cowling, V.H. (2014). Cap-binding complex (CBC). Biochem. J. *457*, 231–242.

Gonatopoulos-Pournatzis, T., Dunn, S., Bounds, R., and Cowling, V.H. (2011). RAM/Fam103a1 is required for mRNA cap methylation. Mol. Cell *44*, 585–596.

Jiao, X., Xiang, S., Oh, C., Martin, C.E., Tong, L., and Kiledjian, M. (2010). Identification of a quality-control mechanism for mRNA 5′-end capping. Nature *467*, 608–611.

Nilson, K.A., Guo, J., Turek, M.E., Brogie, J.E., Delaney, E., Luse, D.S., and Price, D.H. (2015). THZ1 Reveals Roles for Cdk7 in Co-transcriptional Capping and Pausing. Mol. Cell *59*, 576–587.

Perales, R., and Bentley, D. (2009). "Cotranscriptionality": the transcription elongation complex as a nexus for nuclear transactions. Mol. Cell *36*, 178–191.

Pillutla, R.C., Shimamoto, A., Furuichi, Y., and Shatkin, A.J. (1998). Human mRNA capping enzyme (RNGTT) and cap methyltransferase (RNMT) map to 6q16 and 18p11.22-p11.23, respectively. Genomics *54*, 351–353.

Pyronnet, S., and Sonenberg, N. (2001). Cell-cycle-dependent translational control. Curr. Opin. Genet. Dev. *11*, 13–18.

Shafer, B., Chu, C., and Shatkin, A.J. (2005). Human mRNA cap methyltransferase: alternative nuclear localization signal motifs ensure nuclear localization required for viability. Mol. Cell. Biol. *25*, 2644–2649.

Shuman, S. (2015). RNA capping: progress and prospects. RNA *21*, 735–737.

Songyang, Z., Blechner, S., Hoagland, N., Hoekstra, M.F., Piwnica-Worms, H., and Cantley, L.C. (1994). Use of an oriented peptide library to determine the optimal substrates of protein kinases. Curr. Biol. *4*, 973–982.

Stumpf, C.R., Moreno, M.V., Olshen, A.B., Taylor, B.S., and Ruggero, D. (2013). The translational landscape of the mammalian cell cycle. Mol. Cell *52*, 574–582.

Supek, F., Bošnjak, M., Škunca, N., and Šmuc, T. (2011). REVIGO summarizes and visualizes long lists of gene ontology terms. PLoS ONE *6*, e21800.

Tanenbaum, M.E., Stern-Ginossar, N., Weissman, J.S., and Vale, R.D. (2015). Regulation of mRNA translation during mitosis. eLife *4*, e07957.

Topisirovic, I., Svitkin, Y.V., Sonenberg, N., and Shatkin, A.J. (2011). Cap and cap-binding proteins in the control of gene expression. Wiley Interdiscip. Rev. RNA *2*, 277–298.

Tsukamoto, T., Shibagaki, Y., Niikura, Y., and Mizumoto, K. (1998). Cloning and characterization of three human cDNAs encoding mRNA (guanine-7-)-methyltransferase, an mRNA cap methylase. Biochem. Biophys. Res. Commun. *251*, 27–34.

Wen, Y., and Shatkin, A.J. (1999). Transcription elongation factor hSPT5 stimulates mRNA capping. Genes Dev. *13*, 1774–1779.

Wen, Y., and Shatkin, A.J. (2000). Cap methyltransferase selective binding and methylation of GpppG-RNA are stimulated by importin-alpha. Genes Dev. *14*, 2944–2949.

Zaidi, S.K., Young, D.W., Montecino, M.A., Lian, J.B., van Wijnen, A.J., Stein, J.L., and Stein, G.S. (2010). Mitotic bookmarking of genes: a novel dimension to epigenetic control. Nat. Rev. Genet. *11*, 583–589.

Molecular Cell, Volume *61*

Supplemental Information

CDK1-Cyclin B1 Activates RNMT, Coordinating mRNA Cap Methylation with G1 Phase Transcription

Michael Aregger, Aneesa Kaskar, Dhaval Varshney, Maria Elena Fernandez-Sanchez, Francisco A. Inesta-Vaquera, Simone Weidlich, and Victoria H. Cowling

Figure S1

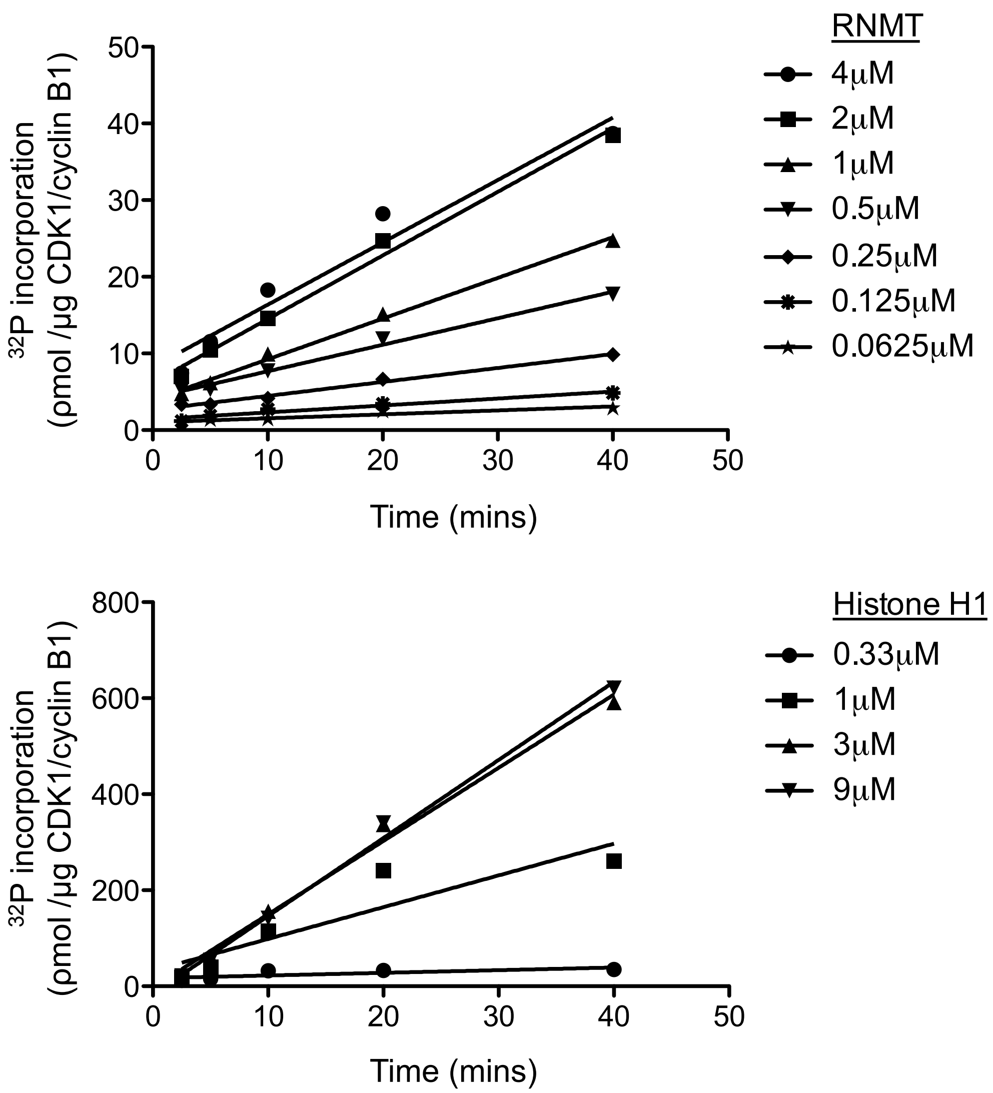

Figure S1, related to Figure 3. CDK-cyclin B1 phosphorylation of RNMT and Histone H1

0.01μM CDK1-cyclin B1 was incubated with a titration of RNMT or Histone H1 and ^{32}P ATP for the time course indicated. Following the kinase reaction, proteins were resolved by SDS-PAGE and ^{32}P-labelled protein was visualised by phosphoimaging and quantified by image densitometry.

Figure S2

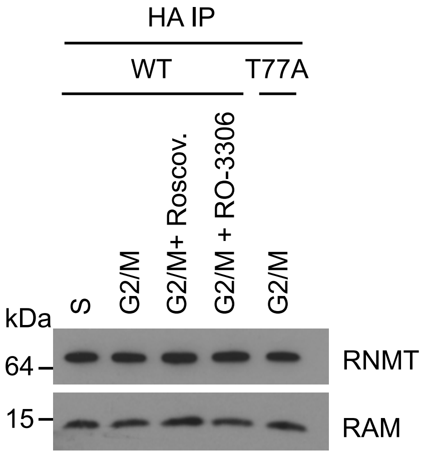

Figure S2, related to Figure 4. RNMT phosphorylation does not impact on interaction with RAM

HeLa cells stably expressing HA-RNMT WT or T77A were synchronised using a double thymidine block and released into normal growth media for 2 hours (S) or 7.5 hours (G2/M), or incubated for 7.5 hours and then for 15 minutes with 50 μM Roscovitine (G2/M + Roscov.) or 9 μM RO-3306 (G2/M + RO-3306). HA-RNMT was immunoprecipitated from 0.6 mg cell extracts using 7 μg anti-HA antibody. Immunoprecipitates were resolved by SDS-PAGE and WBs were performed to detect RNMT and RAM.

Figure S3

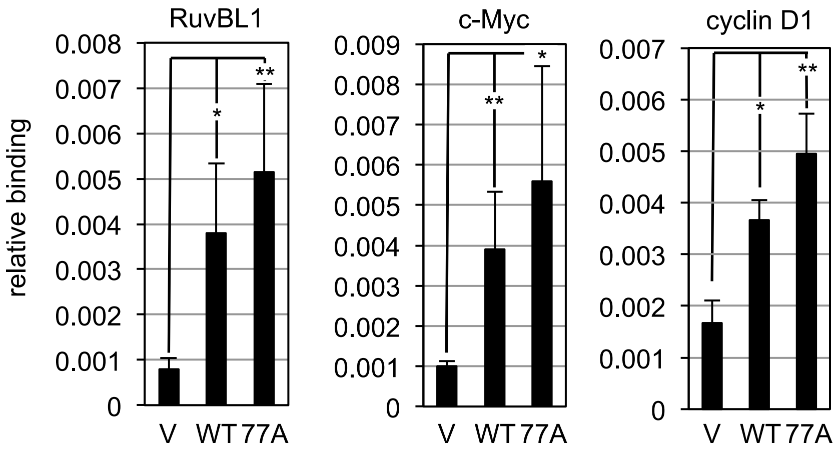

Figure S3, related to Figure 4. Prevention of RNMT T77 phosphorylation does not alter RNMT recruitment to transcription initiation sites

Chromatin immunoprecipitation was performed on HeLa cells transiently transfected with pcDNA4 HA-RNMT WT or T77A or with pcDNA4 (V). Anti-HA antibodies were used to immunoprecipitate RNMT. PCR analysis was performed against promoter proximal regions of the genes indicated. The PCR signal relative to input is depicted for the average of at least three independent experiments and the error bars represent the standard deviation (T-test P-value “*”>0.05, “**”>0.01).

Figure S4

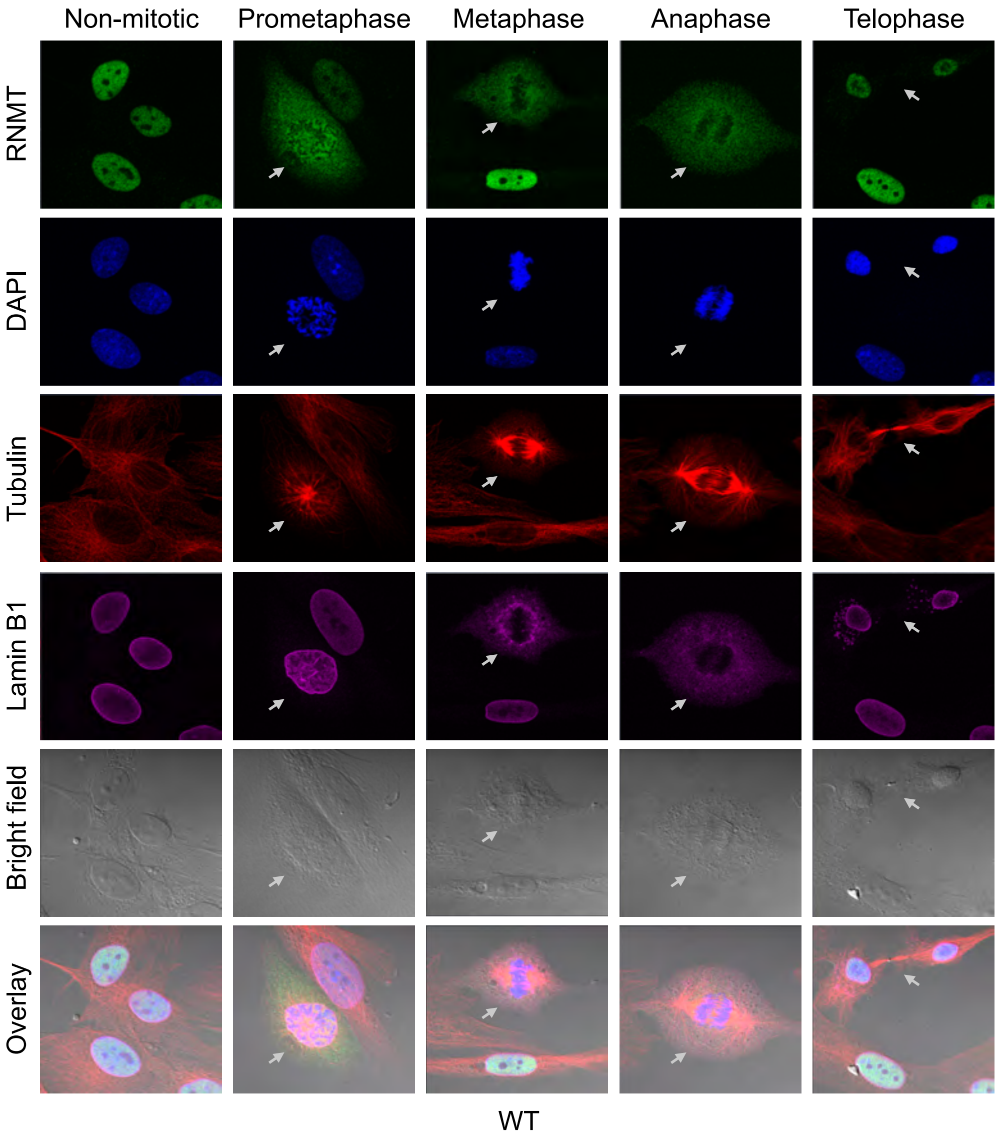

Figure S4, related to Figure 5. RNMT Threonine 77 phosphorylation does not regulate RNMT localisation during mitosis

IMEC cells expressing siRNA-resistant HA-RNMT WT were transfected with siRNMT for 72 hours and immunofluorescence analysis was performed.

Figure S5

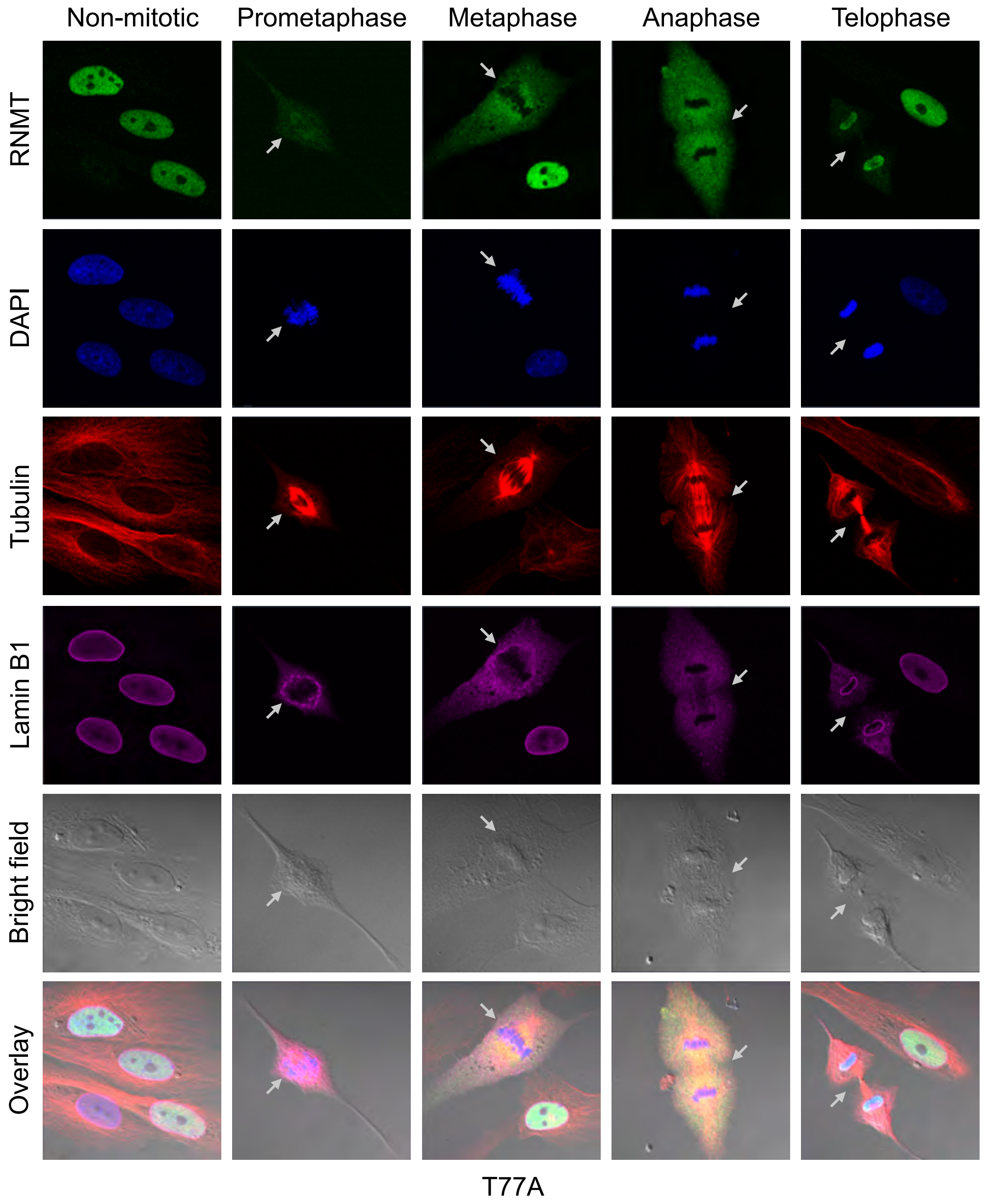

Figure S5, related to Figure 5. RNMT T77 phosphorylation does not regulate RNMT localisation during mitosis

IMEC cells expressing siRNA-resistant HA-RNMT T77A were transfected with siRNMT for 72 hours and immunofluorescence analysis was performed.

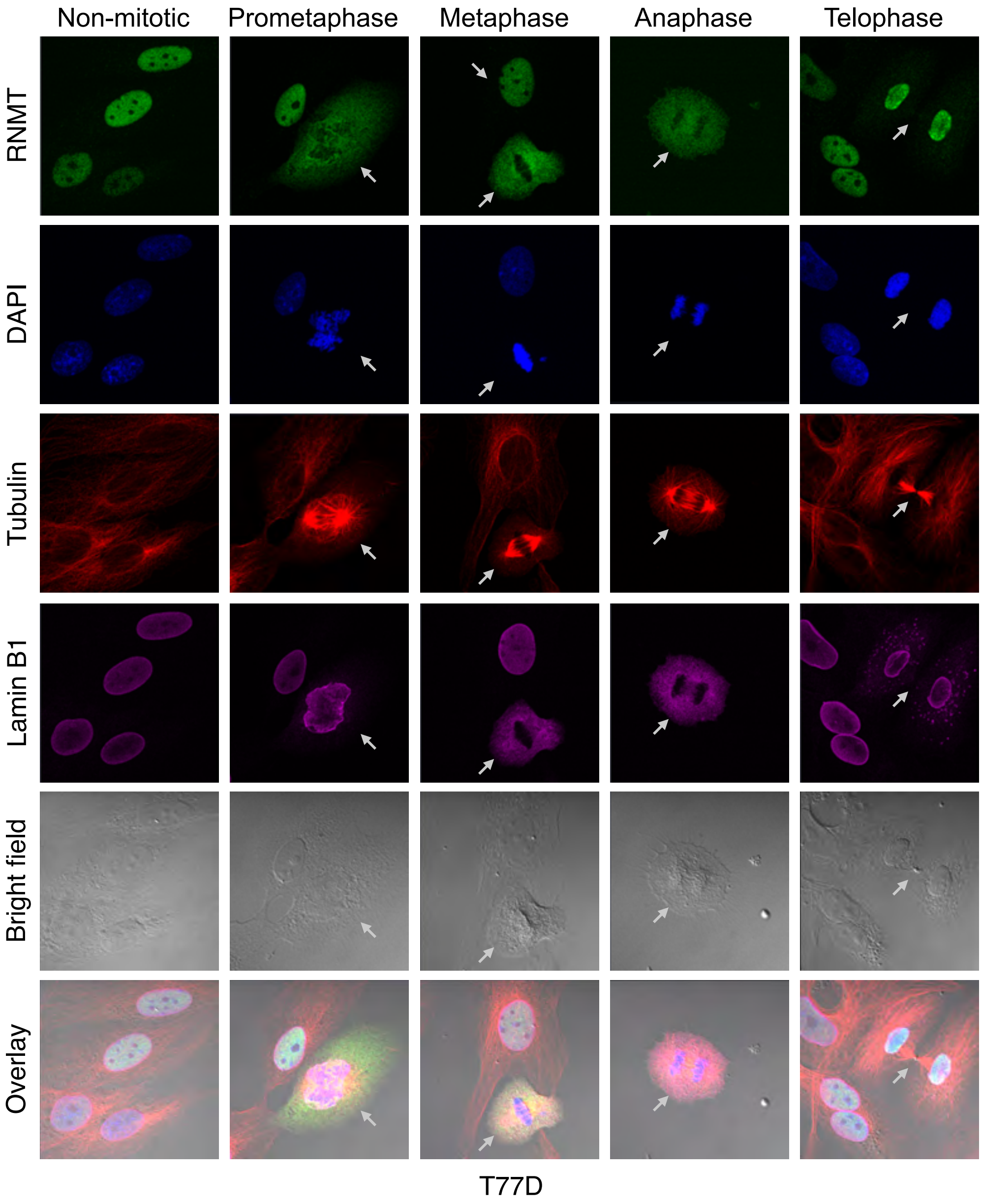

Figure S6, related to Figure 5. RNMT T77 phosphorylation does not regulate RNMT localisation during mitosis

IMEC cells expressing siRNA-resistant HA-RNMT T77D were transfected with siRNMT for 72 hours and immunofluorescence analysis was performed.

Figure S7

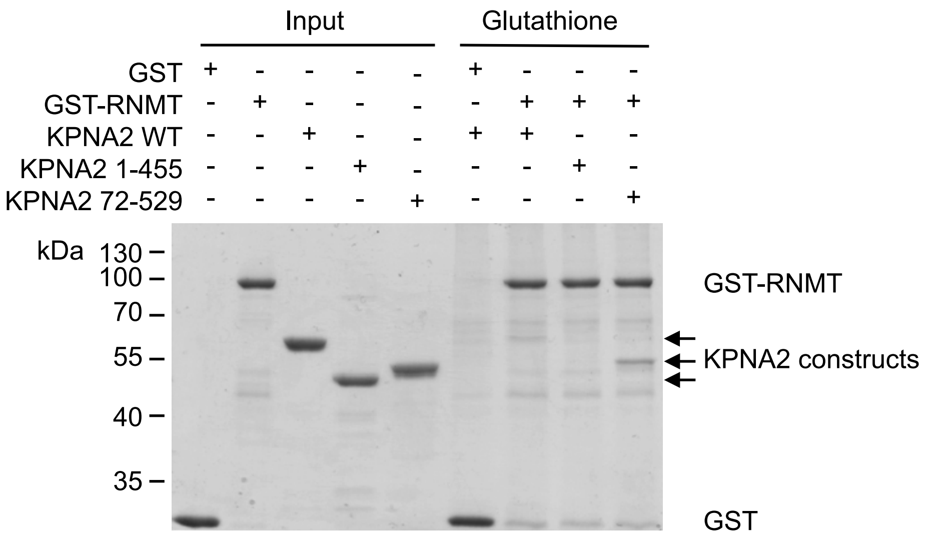

Figure S7, related to Figure 5. RNMT directly interacts with KPNA2 WT and 72-529

Recombinant GST-RNMT and GST were incubated with recombinant KPNA2 WT, 1-455, 72-529 and complexes affinity purified with glutathione-sepharose. Inputs and proteins eluted were resolved by SDS-PAGE and stained by Coomassie blue.

Table S1, related to Figure 6. Summary of proteins identified by Mass spectrometry.
Sequence coverage for mass spectrometry analysis of HeLa cells expressing HA-RNMT WT and HA-RNMT T77A.

Table S2, related to Figure 6. Differential expression analysis of HeLa cells expressing HA-RNMT WT and 77A.
Label Free Quantitation (LFQ) of proteins identified in 3 preparations of HeLa cells expressing HA-RNMT WT and T77A were compared. Proteins that were expressed with a significant difference in the two sample sets are indicated. Ttest P values reported.

Table S3, related to Figure 6. GO Term analysis of proteins expressed significantly differently in response to HA-RNMT WT and 77A.

Supplemental Experimental Procedures

Cell Culture
HeLa and HEK 293 cells were cultured in DMEM/10% Foetal Bovine Serum (GIBCO), in 5% CO_2 at 37ºC. IMEC (Human immortalised mammary epithelial cells) were cultured in DMEM/F-12 HAM supplemented with L-glutamine, 5 µg/ml insulin, 10 ng/ml EGF and 0.5 µg/ml hydrocortisone. Stable cell lines expressing INI (vector control) and INI-HA-RNMT constructs were generated by retroviral infection using standard protocols and selected with 0.5 mg/ml G418. siRNAs and controls were purchased from the siGenome range (Dharmacon). 3 µl Gene Juice (Novagen) was used to transfect 1.2 x10^5 cells with 1 µg DNA, 4 µl Lipofectamine RNAiMax (Invitrogen) was used to transfect 1.2 x10^5 cells with 4 µl 50µM siRNA, and Lipofectamine 2000 (Invitrogen) was used to simultaneously transfect 2.5 x10^5 cells with 4 µl siRNA and 1 µg DNA. Cells were lysed in Triton lysis buffer (10 mM Tris [pH 7.05], 50 mM NaCl, 50 mM NaF, 10% glycerol, 0.5% Triton X-100, protease and phosphatase inhibitors (Sigma)). Kinase inhibitors used were Roscovitine (Sigma), RO-3306 (Merck), DRB (Sigma) and Flavopiridol (Cayman). Soft agar assay: 5 x 10^5 IMEC cells were plated in 0.3% noble agar/5% serum/growth medium and fed every 2 days.

Orthophosphate Labelling
HEK 293 cells were washed with PBS, incubated at 37°C in phosphate-deficient DMEM/10% dialysed FBS for 4 h,then labelled with 0.03 mCi/ml ^{32}P orthophosphate for 2 h. 2.5 mg cell extract was precleared for 30 min with 30 µl protein A/G PLUS agarose and HA-RNMT immunoprecipitated for 4 h using 10 µg monoclonal anti-HA antibody-conjugated agarose (Sigma). Immunoprecipitates were washed with Triton lysis buffer and resuspended in 50 µl Lamelli buffer. 24 µl was resolved by SDS-PAGE and analysed by autoradiography and 5 µl was used for western blot analysis.

Immunoprecipitation
Performed at 4ºC. RNMT phosphorylation analysis: RNMT was immunoprecipitated from 600 µg HeLa cell extracts for 2.5 hours, using 6 µg mouse monoclonal anti-HA antibody-conjugated agarose or 1.5 µg sheep polyclonal anti-RNMT plus 20 µl Protein A/G agarose. Immunoprecipitates were washed and resolved by 8% SDS-PAGE. 50% of immunoprecipitates were used to detect pT77 RNMT and 10% used to detect RNMT. Other immunoprecipitations were performed with 1.5µg antibody and 1-5mg cell extract.

m7G immunoprecipitation
Experiments performed essentially as in (Cole and Cowling, 2009). HeLa cells were transfected with pcDNA5 HA-RNMT WT, 77A or vector control for 24 hrs. RNA was extracted and repurified by phenol:chloroform extraction. 5 µg was subject to immunoprecipitation with 10 µl anti-m7G antibody or polyclonal control rabbit antibody for 1 hr at room temperature. Immunoprecipitates were washed three times and resultant RNA used as a template for RTPCR.

Peptide pull down
Performed at 4 °C. 10 mg cell extracts were precleared five times for 10 minutes with 20 µl 50 % streptavidin-sepharose, incubated with 1-3 µg biotinylated peptides for 10 minutes and 20 µl 50 % streptavidine-sepharose for 5 minutes. Sepharose was washed three times with Triton Lysis buffer, proteins resolved by SDS-PAGE and identified by western blot or peptide mass fingerprinting. Protein was trypsinised and peptides resolved by nanoLC system coupled to LTQ-Orbitrap mass spectrometer. Results were searched against the SwissProt or IPI human databases using the Mascot Daemon. Mass Spec performed by MRC PPU mass spectrometry team.

GST pull down
Recombinant proteins including GST fusions were incubated for 3 hours at 4 °C, affinity purified with 25 µl 50% slurry glutathione-sepharose, washed five times with Triton Lysis Buffer and resolved by SDS-PAGE.

Flow cytometry analysis
HeLa cells were fixed by dropwise addition of cold Ethanol to 70% concentration. Cells were washed twice in PBS/1 % FBS and stained in PI solution (PBS, 1 % FBS, 50 µg/ml RNase A, 50 µg/ml propidium iodide) for 30 minutes at room temperature in the dark. DNA content was assessed using a FACS Calibur flow cytometry (Becton Dickinson). 10,000 events were counted per sample and DNA content was plotted on a histogram from channel number 0 to 1023 using the FlowJo software. The G1 peak was set to 200 to allow resolution of the G1, S and G2/M phases. Cell cycle distribution was estimated with FlowJo using the Watson distribution model

Immunofluorescence analysis

IMEC were seeded on cover slips and fixed with 4% paraformaldehyde in PHEM for 7 minutes at 37°C. Cells were permeabilised with 0.02 % Triton X-100 in TBST for 2.5 minutes and blocked with 10 % Donkey serum in antibody dilution solution (PBS, 0.2% BSA, 0.02% sodium azide) for 30 minutes. Cells were incubated with anti-HA (in house, 1:2,000), anti-Tubulin (Abcam,1:500) and anti-Lamin (Abcam, 1:1,000) antibodies for 1 hour at room temperature. Subsequently, cells were incubated with Alexa Fluor 488 Donkey anti-Sheep, Alexa Fluor 594 Donkey anti-Rat and Alexa 642 Donkey anti-Rabbit secondary antibodies (all Invitrogen, 1:500) for 50 minutes in the dark and stained with DAPI (Invitrogen, 1:50,000). Fluorescence microscopy was performed on a Zeiss LSM 700.

Western blotting

Western blots were performed according to standard protocols. Antibodies were raised against recombinant human RNMT, human RAM, and GST in sheep and affinity purified. Antibodies also used: monoclonal anti-HA (Sigma), and anti-FLAG (Sigma) antibodies, and polyclonal anti-Tubulin (Santa Cruz), Cyclin B1 (CST), Histone 3 pS10 (Abcam), Cyclin E1/2 (SCBT), Cyclin A2 (SCBT), CDK1 (CST), CDK3 (BIOSS), β-Tubulin (Santa Cruz), Tomm70A (Insight), Bop1 (Insight), DDX18 (Insight), Skp1 (SCBT) and KPNA2 (Thermo) antibodies. RNMT Phospho-T77 antibody production: antibodies were raised in sheep against the phosphopeptide RNMT 73CGKDT*PSKKR82 (T* indicates phospho-threonine) coupled separately to keyhole limpet hemocyanin and bovine serum albumin. Sera were purified by affinity chromatography on immobilized peptide antigen.

Chromatin immunoprecipitations (ChIP)

Chromatin immunoprecipitations were performed using the Millipore ChIP kit according to manufacturer's instructions. 15 μg anti-HA-agarose (Sigma) was used to immunoprecipitate HA-RNMT. The DNA was purified with a QIAquick PCR purification kit (Qiagen) and eluted in 50 μl distilled water. 2 μl of input and immunoprecipitated DNA were subjected to RT-PCR analysis. ChIP signal was determined relative to input.

RTPCR analysis

RTPCR performed with a BioRad iQ5 RT-PCR detection system using Quanta Biosciences SYBR Green FastMix for iQ and the primers listed below. Primers used were:

BOP1	GTGGGCTTCAACCCCTATGAG	CCATGCGAGAGACCTTCTCC
Tomm70A	CCATATACCTGTGGAGTCGGC	GAGAGTTCATGTCCAAGTGAGCA
DDX18	GGAAGGCAGGGATCTTCTAGC	TCCATTCCTGGGCATGAACC
Skp2	ATGCCCCAATCTTGTCCATCT	CACCGACTGAGTGATAGGTGT
RuvBL1-chIP	TGTGGCCAGTGGACC	ACTTCCCTGAGGAAATAATGG
c-Myc-chIP	GCACTGGAACTTACAACACC	ATCCAGCGTCTAAGCAGC
cyclin D1-chIP	AGCTGCCCAGGAAGAGC	CCGCCTTCAGCATGG

Proteomic analysis

HeLa cells were lysed in 8M urea/100 mM Tris-HCl (pH 8.0)/protease and phosphatase inhibitors (Roche), 10mM DTT added for 30 mins at 30°C, then 50 mM carboxyamidomethylated iodoacetamide for 45 mins at room temperature in dark. Urea concentration was adjusted to 0.8 M in Tris/$CaCl_2$ buffer before adding Trypsin Gold (Wako) to a substrate: trypsin ratio of 100:1 (w/w), and incubated at 30°C overnight. Peptides were separated into 10 fractions on hydrophilic strong anion exchange column and analysed on Ultimate 3000 RSLCnano system coupled to a LTQ OrbiTrap Velos Pro (both Thermo Scientific). MaxQuant was used to analyse RAW data using *H. sapiens* Uniprot database using a false discovery rate of 1%. Contaminant, reverse and only identified by site entries were removed. For the three replicates, average LFQ intensity and Student T-test P-value was calculated (unpaired, two-tailed, unequal variance). Gene ontology analysis was carried out using REVIGO (Supek et al., 2011).
